# Novel Analytical Workflow for Tracing Synthetic Caffeine Adulteration in Amazonian Guaraná (*Paullinia cupana*) Using Low-Field ^1^H NMR Fingerprinting and Chemometrics

**DOI:** 10.1021/acs.jafc.6c08341

**Published:** 2026-08-10

**Authors:** Jelmir Craveiro de Andrade, Pedro Lucas Caetano de Oliveira, Maxsueli Machado, Adriano Teixeira de Oliveira, Adriene Núzia de Almeida Santos, Carlos Adam Conte-Junior

**Affiliations:** † Center for Food Analysis (NAL), Technological Development Support Laboratory (LADETEC), Federal University of Rio de Janeiro (UFRJ), Cidade Universitária, Rio de Janeiro 21941-598, RJ, Brazil; ‡ Laboratory of Advanced Analysis in Biochemistry and Molecular Biology (LAABBM), Department of Biochemistry, Federal University of Rio de Janeiro (UFRJ), Cidade Universitária, Rio de Janeiro 21941-909, RJ, Brazil; § Federal University of Mato Grosso (UFMT), Cuiabá 78060-900, MT, Brazil; ∥ Center for Studies of Invertebrates and Vertebrates of the Amazon (NEIVA), Federal Institute of Education, Science and Technology of Amazonas (IFAM), Manaus Centro Campus (CMC), Manaus 69020-120, AM, Brazil; ⊥ Graduate Program in Animal Science and Fisheries Resources (PPGCARP), Faculty of Agricultural Sciences (FCA), Federal University of Amazonas (UFAM), University Campus, Manaus 69077-000, AM, Brazil; # Graduate Program in Veterinary Sciences (PPGCVET), Federal Institute of Education, Science and Technology of Amazonas (IFAM), Nilton Lins University Center (CUNL), Manaus 69020-120, AM, Brazil; ∇ Graduate Program in Biodiversity and Science Education in the Amazon (PPGBEC), Federal Institute of Education, Science and Technology of Amazonas (IFAM), Amazonas State University (UEA), Manaus 69020-120, AM, Brazil; ○ Amazonas State University, Manaus 69050-010, AM, Brazil; ◆ Nilton Lins University Center (CUNL), Manaus 69058-020, AM, Brazil

**Keywords:** guaraná, food authentication, geographical indication, DD-SIMCA

## Abstract

Maués guaraná (*Paullinia cupana*), a Brazilian product with Geographical Indication (GI), is highly susceptible to economically motivated fraud, particularly through the undeclared addition of synthetic caffeine. Conventional chromatographic methods for authenticity control are time-consuming, costly, and rely on hazardous chemicals. This study presents a comparatively streamlined, solvent-efficient, and environmentally friendly approach based on low-field ^1^H NMR spectroscopy coupled with chemometrics, eliminating the need for deuterated solvents. Using authentic (*n* = 40), commercial (*n* = 18), and adulterated samples (1–10% synthetic caffeine), the data-driven soft independent modeling of the class analogy (DD-SIMCA) model achieved 100% sensitivity and over 99% accuracy for authentication. Simultaneously, a PLS regression model accurately quantified adulteration levels, showing high predictive performance (*R*_pred_^2^ = 0.9891; RMSEP = 0.36%) and low detection limits. This orthogonal workflow successfully distinguished natural agro-industrial variability from intentional adulteration, demonstrating the potential of a practical, affordable, and sustainable first-line screening approach for future regulatory application.

## Graphical abstract

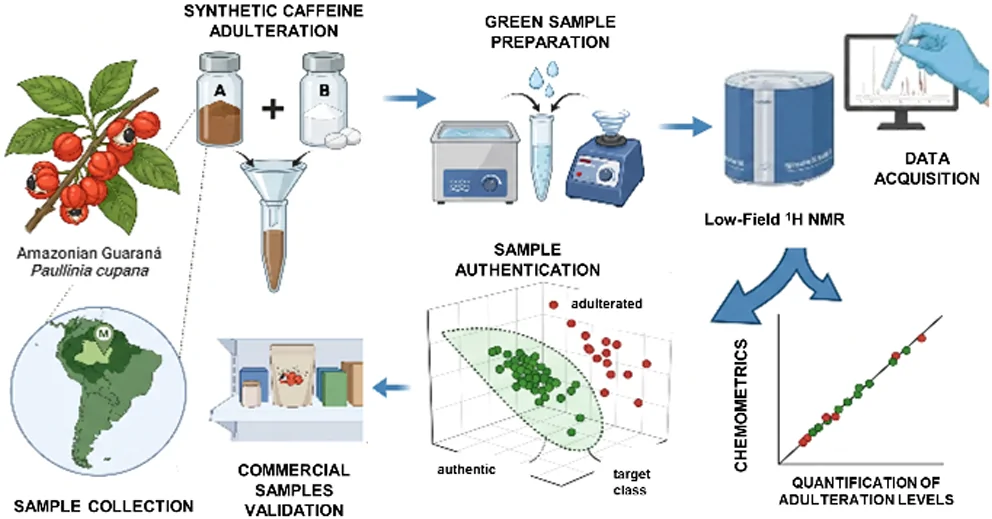

## 1 Introduction

Guaraná (*Paullinia cupana* Kunth) holds a strategic position in the global scientific and industrial landscape, with applications ranging from dietary supplements and energy drinks to pharmaceutical and cosmetic formulations. Traditionally cultivated in the Amazon region, guaraná has been used for centuries by indigenous populations.^1,2^ From a botanical and chemical perspective, guaraná is notable for its high methylxanthine content. Caffeine (1,3,7-trimethylxanthine), a primary bioactive alkaloid, can vary between 2 and 6% of dry mass, concentrations significantly higher than those found in other classic botanical sources such as coffee and tea.^3,4^ In this context, the municipality of Maués, in the state of Amazonas, has established itself as the birthplace of guaraná in the world. It holds a Geographical Indication (GI) of Origin, which certifies the excellence, typicality, and traceability of its cultivation. This seal guarantees a product with a unique chemical signature, deeply influenced by the Amazonian terroir and traditional agro-industrial processing methods.^1,5^

The growing demand for natural and functional products has driven the use of the guaraná extract as a highly valued source of natural energy. The physiological effects, which include increased alertness, improved cognitive performance, and metabolic support for athletic activities, result from the synergistic action of caffeine with other intrinsic seed constituents, such as theobromine, theophylline, tannins, and saponins.^4,5^ However, the high bioactivity of guaraná demands extremely rigorous quality and dosage control. Inappropriate doses of caffeine can negate therapeutic benefits and trigger severe adverse effects, such as tachycardia, anxiety, and insomnia, representing critical risks for sensitive populations like pregnant women, children, and individuals with cardiovascular conditions.^6,7^ For this reason, regulatory agencies impose strict limits on the addition and consumption of caffeine in commercial formulations.

In Brazil, the National Health Surveillance Agency (ANVISA) stipulates that dietary supplements must provide between 75 and 200 mg of caffeine per day for the adult population. The maximum limit of 400 mg daily is exclusive for athletes, provided that this amount is divided into doses of up to 200 mg. For energy drinks, the agency restricts the maximum concentration to 35 mg per 100 mL.^8^ This national regulatory rigor converges with the safety guidelines of international institutions, such as the European Food Safety Authority (EFSA) and the Food and Drug Administration (FDA).^9,10^ Despite these strict regulations, the high commercial value of natural guaraná combined with the low cost of synthetic caffeine creates a lucrative scenario for economically motivated adulteration. Fraudsters deliberately spike low-grade or exhausted guaraná matrices with synthetic caffeine to mimic the natural alkaloid profile, deceiving standard quality control protocols and potentially exposing consumers to unregulated doses. Consequently, this highlights the global need for precise and robust analytical methods capable of detecting such sophisticated adulterations to certify the compliance and safety of these products.^11,12^

Despite regulatory requirements, the guaraná production chain still faces challenges regarding fraud through adulteration. Globally, economically motivated adulteration is an escalating threat; reports from the FDA and USP estimate that food and botanical fraud affects approximately 1% of the global industry, costing up to $40 billion annually.^13–15^ This issue is particularly critical for guaraná from Maués. Due to its certification of origin, this product carries high added value in the market, making it an attractive target for counterfeiters seeking higher profits.^1,5^ To bypass price fluctuations and reduce operational costs, the intentional and undeclared addition of synthetic caffeine (anhydrous and pharmaceutical grade) to guaraná extracts and powders has become a recurring fraudulent practice, where they are marketed as purely natural products.^7^ Although natural and synthetic caffeine are chemically identical molecules, illicit fortification compromises the integral chemical complexity of the natural matrix.^5,7^ The differentiation between the authentic product and the falsely enriched one has become a topic of extreme regulatory relevance. Consumers and oversight bodies demand rigorous authenticity control, requiring methodologies capable of not only quantifying caffeine but also certifying the authenticity and origin of the plant matrix, thereby protecting high-value products like the certified guaraná from Maués.^2,16^

Traditionally, the quality assessment of guaraná and the determination of its caffeine content have relied heavily on chromatographic techniques. High-Performance Liquid Chromatography (HPLC) coupled with UV detection remains the most widely adopted method and is recommended by official compendia. Furthermore, hyphenated approaches, particularly Liquid Chromatography-tandem Mass Spectrometry (LC-MS/MS), represent the analytical gold standard for structural elucidation and ultratrace analysis owing to their high sensitivity and resolution.^5,16^ However, the routine application of these advanced techniques for high-throughput regulatory screening is frequently limited by operational constraints. These methods generally require substantial infrastructure, high consumption of organic solvents, and extensive sample-preparation steps, such as repetitive extractions and solid-phase extraction (SPE), to mitigate matrix interferences caused by polyphenols and saponins. Moreover, while conventional chromatographic protocols are highly precise for targeted analyte quantification, extending these methods to untargeted profiling to detect matrix perturbations demands complex workflows that may not be feasible for rapid, large-scale sample screening.^17,18^

To address the increasing sophistication of economically motivated adulteration, low-field proton nuclear magnetic resonance (LF ^1^H NMR) spectroscopy has emerged as an environmentally favorable platform for rapid spectral acquisition and high-throughput screening.^19,20^ Its suitability for limited sample manipulation is strongly matrix-dependent. While homogeneous liquid samples may often be analyzed directly, heterogeneous and particulate botanical materials generally require standardized extraction and clarification to obtain representative, particle-free solutions suitable for reproducible spectroscopic analysis.^21^ From an economic perspective, LF NMR offers substantial advantages over conventional high-field instrumentation. High-field spectrometers typically require major capital investment, cryogenic liquids, and dedicated shielded facilities, whereas benchtop low-field instruments, generally costing approximately USD 80,000–120,000, employ permanent magnets that operate at room temperature. Consequently, they avoid cryogenic maintenance and can reduce both infrastructure requirements and total operating costs.^22^ However, important technical limitations must be acknowledged. The lower magnetic field strength inherently reduces spectral resolution and increases signal overlap, particularly in complex botanical matrices. As a result, direct visual interpretation and simple univariate quantification from raw LF NMR spectra are often insufficient, making standardized sample preparation, spectral preprocessing, and multivariate chemometric analysis essential for reliable authentication and quantification.^23^

To effectively mitigate these inherent analytical bottlenecks, the application of robust data processing and multivariate chemometric workflows is strictly required. By mathematically extracting latent chemical information from highly overlapped spectral profiles, chemometrics significantly expands the diagnostic capability of low-field NMR.^21,24,25^ Pattern recognition through one-class modeling, such as data-driven soft independent modeling of class analogy (DD-SIMCA), enables comprehensive characterization of the natural variability within the authentic matrix. This approach establishes statistical boundaries that are highly sensitive to metabolic deviations, functioning as a reliable method for authenticity screening.^24,26^ Concurrently, predictive algorithms such as partial least-squares regression (PLSR) allow for the precise quantification of specific adulteration markers, successfully converting the rapid fingerprinting capacity of LF NMR into a highly specific and quantitative regulatory tool.^21,24^

In this context, the present study aimed to develop and validate a rapid and interpretable LF ^1^H NMR-based method for authenticating and quantifying synthetic caffeine addition in guaraná powder. The proposed workflow combines standardized aqueous extraction, LF NMR fingerprinting, and chemometrics without the routine use of deuterated or organic solvents. Consequently, it provides a comparatively streamlined, solvent-efficient, and accessible screening strategy with favorable alignment with the Analytical GREEnness (AGREE) criteria, particularly through the reduced energy requirements, replacement of hazardous and deuterated solvents with ultrapure water, and improved operator safety.

## 2 Experimental Section

### 2.1 Sampling and Sample Preparation

#### 2.1.1 Sampling Sites and Sample Collection

The sampling strategy was designed to ensure the botanical authenticity and representativeness of the guaraná samples, thereby supporting the construction of robust chemometric models for fraud identification. For the reference group, 40 authentic guaraná powder samples were obtained directly from producers at three farms located in the municipality of Maués, Amazonas: Farm 1 (3.389°S, 57.686°W; *n* = 9), Farm 2 (3.386°S, 57.693°W; *n* = 15), and Farm 3 (3.403°S, 57.673°W; *n* = 16). To preserve the anonymity of the producers, the geographical coordinates are reported with reduced spatial precision. This region holds a Geographical Indication (GI) certification under the Indication of Source modality, recognized by the Brazilian National Institute of Industrial Property (INPI), which attests to the geographical origin and preservation of traditional production practices. To capture the biological variability of the species, samples were collected from different individual plants at each farm. Processing followed the regional artisanal protocol, comprising manual harvesting, washing, controlled roasting, and seed grinding, resulting in a 100% natural product without added ingredients. Complementarily, 18 commercial samples were purchased from local markets for evaluation under real-market conditions, as illustrated in Figure 1.

**Figure 1:**
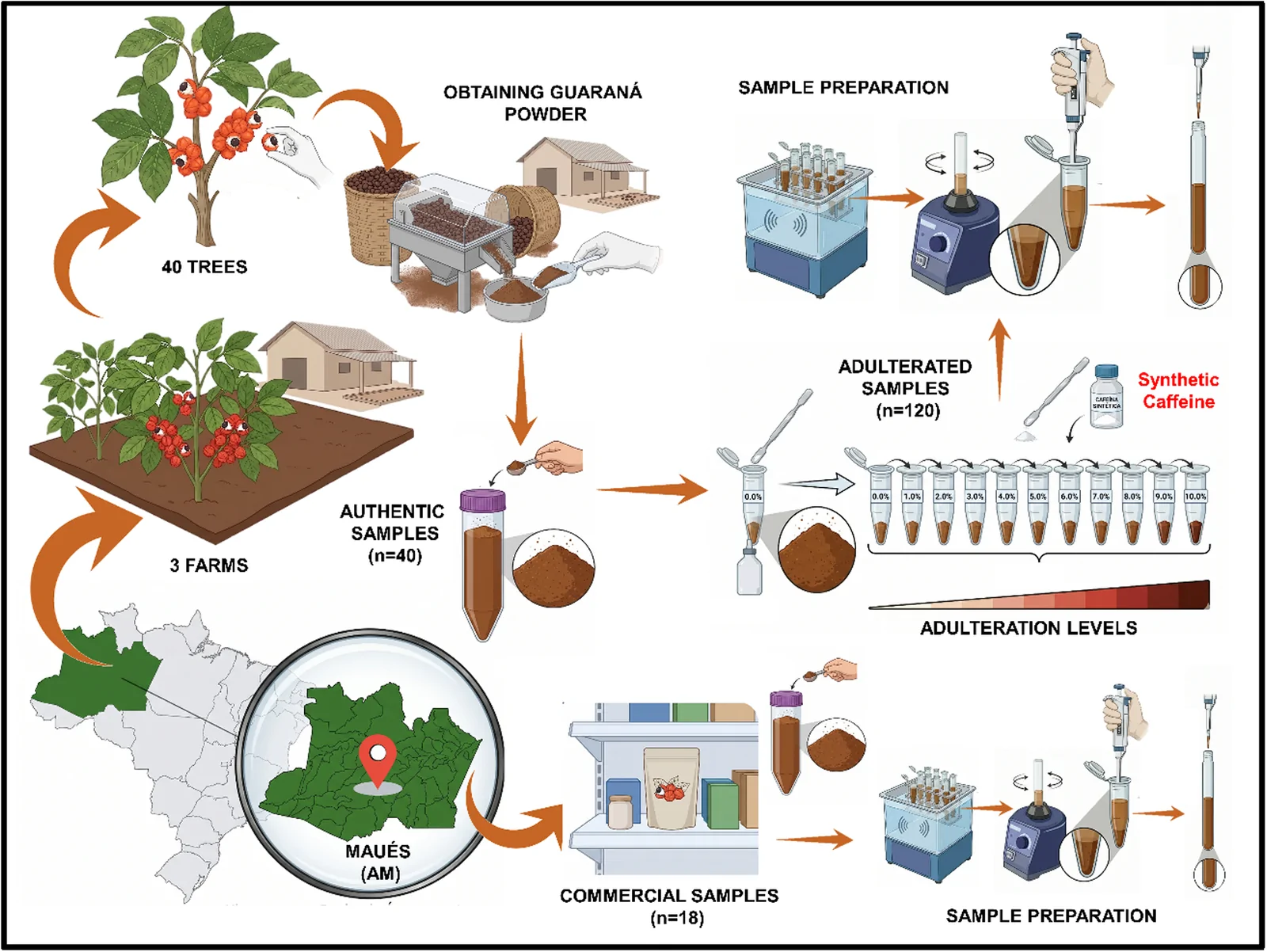
Representative scheme of sample origin and processing, showing the collection of guaraná seeds from Maués-AM and the powder obtained through the traditional method of artisanal roasting and grinding. The flow illustrates the preparation steps for authentic samples and the spiking strategy to obtain adulterated samples with different levels of synthetic caffeine without the use of deuterated solvents.

#### 2.1.2 Preparation of Laboratory-Adulterated Samples

For the adulteration simulation, commercially available caffeine capsules, purchased from pharmacies, were used as the source of synthetic caffeine. According to the product label, caffeine was the only ingredient listed. Before sample preparation, several capsules were opened, and their powder contents were pooled and thoroughly homogenized to obtain a uniform material. The homogenized capsule powder was used directly, without additional extraction or purification. Laboratory-adulterated samples were prepared gravimetrically on a final-mixture-mass basis. For all preparations, the combined mass of authentic guaraná powder and synthetic caffeine was maintained at 100 mg. Accordingly, the 0% control consisted of 100 mg of authentic guaraná powder without added caffeine, whereas samples containing 1–10% (w/w) synthetic caffeine were prepared by combining 1–10 mg of the homogenized caffeine powder with 99–90 mg of authentic guaraná powder, respectively. Four authentic samples originating from different individual plants were selected from each of the three farms, resulting in 12 distinct authentic matrices. Each matrix was independently prepared at each of the ten addition levels, generating 12 samples per level and 120 laboratory-adulterated samples in total. All samples were independently weighed, spiked, extracted, and analyzed. The detailed gravimetric composition and number of preparations are provided in Table S1.

The 1–10% (w/w) range was established as an experimental calibration interval rather than as a regulatory threshold or a direct estimate of the adulteration levels occurring in commercial products. Its selection considered both the reported variability in caffeine concentrations among guaraná products and the need to evaluate the analytical performance of the proposed method across progressively increasing addition levels. Caffeine concentrations of approximately 9.52–36.71 mg g^–1^, corresponding to approximately 0.95–3.67% (w/w), have previously been reported in guaraná products.^6,7^ Therefore, the addition of 1% (w/w) synthetic caffeine, equivalent to 10 mg g^–1^, represents an increase of approximately 27–105% relative to this reported concentration range and was selected as a comparatively low and analytically challenging addition level. The upper level of 10% was included to provide a broad calibration interval and to evaluate model linearity, response stability, and predictive performance under more pronounced fortification conditions.

#### 2.1.3 Extraction and Preparation for LF ^1^H NMR Analysis

The present workflow was designed for a heterogeneous and particulate botanical matrix, making direct solution-state LF ^1^H NMR analysis of guaraná powder unsuitable. Therefore, the extraction procedure was adapted from previously described methodologies.^4,27^ Following gravimetric preparation, 1000 μL of an aqueous solution containing 0.05% (w/v) TMSP was added to each 100 mg sample. The extraction solution was prepared by dissolving 50 mg of TMSP in ultrapure Milli-Q water and adjusting the final volume to 100 mL, corresponding to a TMSP concentration of 0.5 mg mL^–1^. TMSP was used exclusively for chemical-shift referencing and spectral alignment and was not employed as an internal quantitative calibrant. No buffer or pH adjustment was applied in order to preserve the native aqueous spectral fingerprint, avoid changes in ionic strength and matrix interactions, and maintain a comparatively streamlined sample-preparation procedure.

Authentic, laboratory-adulterated, and commercial samples were subjected to the same extraction procedure. Briefly, each 100 mg sample was vortex-homogenized for 1 min and subjected to ultrasound-assisted extraction for 30 min at 25 °C. After centrifugation at 25,155*g* for 5 min, a 500 μL aliquot of the supernatant was transferred to a 5 mm NMR tube for LF ^1^H NMR acquisition. Commercial samples were analyzed without experimental caffeine addition. To minimize preparation-related variability, sample mass, extraction-solution volume, homogenization time, ultrasound conditions, centrifugal force, centrifugation time, and transferred supernatant volume were kept constant for all sample groups.

### 2.2 LF ^1^H NMR Analysis

The ^1^H NMR spectra were acquired using a 60 MHz Spinsolve Multi X spectrometer (Magritek GmbH) equipped with a 1.5 T permanent magnet and an automated sampler. For each analysis, 500 μL of the extract was transferred to a 5 mm NMR tube. Prior to acquisition, samples were thermally equilibrated for 10 min within the spectrometer’s internal chamber (26.5–28.5 °C). This equilibration step was included to minimize temperature-dependent variations in chemical shifts and signal profiles, thereby improving spectral comparability among samples before chemometric analysis. The spectra were collected using a 1D PROTON sequence with a 90° flip angle (pulse length of 12.5 μs) and active ^13^C decoupling to eliminate heteronuclear couplings. A total of 128 scans were accumulated with a spectral width of 5 kHz and 32,768 data points, resulting in an acquisition time of 6.4 s. The repetition time between scans was set to 7 s, totaling approximately 15 min of acquisition per sample. The system was automatically calibrated every 2 h using a H_2_O/D_2_O (10:90 v/v) mixture to ensure field stability. To quantitatively evaluate the environmental impact of the proposed analytical workflow, the Analytical GREEnness (AGREE) metric approach was applied.^28^ The method’s performance was assessed against the 12 principles of Green Analytical Chemistry (GAC). This LF 1H NMR methodology showed favorable alignment with the principles evaluated by the AGREE metric, particularly Criterion 6, by avoiding derivatization; Criterion 9, by reducing energy requirements through the use of a permanent-magnet spectrometer without cryogenic cooling; Criterion 11, by replacing hazardous and deuterated solvents with ultrapure water; and Criterion 12, by improving operator safety. The AGREE assessment yielded an overall score of 0.68, and the corresponding pictogram and score distribution are provided in the Supporting Information, supporting the favorable greenness profile and solvent efficiency of the proposed screening workflow.

### 2.3 Multivariate Analysis

All stages of data preprocessing, multivariate analysis, and postprocessing were performed using a hybrid computational approach. Unsupervised exploratory chemometric modeling was conducted using the γ-GUI graphical interface (version 2.0, Multivariate Analysis in Food Matrices Group - γ, Brazil, 2024), an application developed by our research group.^29^ This tool operates on the MATLAB 2023a platform (The MathWorks Inc., Natick, Massachusetts, USA) and is freely available for download in the GitHub repository (https://github.com/appGAMMA). Complementarily, customized algorithms, including the DD-SIMCA approach originally proposed by Zontov et al. (2017),^30^ and processing pipelines were developed in Python (version 3.13.1, Python Software Foundation, Wilmington, Delaware, USA). The computational workflow incorporated an ecosystem of open-source libraries for scientific computing and machine learning. Data organization and structural manipulation were performed using Pandas (version 2.2.3, NumFOCUS, Austin, Texas, USA) and NumPy (version 2.1.3, NumFOCUS, Austin, Texas, USA). Statistical operations, Euclidean-distance calculations, and probability-distribution functions were implemented using SciPy (version 1.15.2, NumFOCUS, Austin, Texas, USA). Partial least-squares regression routines and model-validation metrics were implemented using Scikit-learn (version 1.6.1, Inria, Saclay, France). Data visualization and figure generation were performed using Matplotlib (version 3.10.0, 2024, NumFOCUS, Austin, Texas, USA).^31^

In this study, supervised chemometric approaches were employed to ensure the integrity of guaraná powder and detect fraud involving the addition of synthetic caffeine. Based on this spectral matrix, three modeling approaches were conducted to meet the study’s objectives: (i) Authentication; (ii) Classification by adulteration levels; and (iii) Quantification of adulteration. For the authentication task, the DD-SIMCA method was utilized to rigorously distinguish authentic samples from adulterated ones. Complementarily, aiming not only at detection but also at quantifying adulteration levels, PLSR was applied. While DD-SIMCA focused on verifying sample compliance, the PLSR model was developed to quantify the level of synthetic caffeine adulteration. This multivariate calibration technique correlates variations in the intensities of ^1^H NMR spectral signals with the known concentrations of added synthetic caffeine, allowing for a precise estimation of the adulterant content in commercial and validation samples.

#### 2.3.1 Spectral Corrections

The free induction decay (FID) signals underwent a standardized processing workflow before chemometric analysis. A 0.5 Hz exponential line-broadening function was applied during spectral processing to improve the signal-to-noise ratio. Phase and baseline corrections were subsequently performed to obtain absorptive peak shapes and minimize instrumental drift and background variation. Spectral alignment was carried out using the TMSP resonance at δ 0.00 ppm. After alignment, the TMSP signal was excluded from the chemometric data matrix to prevent the reference compound from contributing to the multivariate models.

The spectral interval between δ 0.00 and 9.50 ppm was retained. No water-signal suppression technique was applied during data acquisition; instead, the residual water-resonance region between δ 4.50 and 5.00 ppm was digitally excluded before normalization and chemometric modeling. No other spectral regions were removed. These exclusions were consistently applied to principal component analysis (PCA), DD-SIMCA, and PLSR; therefore, discontinuities in the corresponding loading, discriminatory power, and variable importance in projection (VIP) profiles reflect the removed spectral intervals.

Three normalization strategies were evaluated to minimize variations associated with sample extraction, dilution, and overall signal intensity, maximum normalization (norm ∞), sum-of-absolute-values normalization (norm 1), and Euclidean normalization (norm 2). Following preprocessing and spectral-region exclusion, the common master matrix comprised 178 LF ^1^H NMR spectra and 6.891 spectral variables. The data set included 40 authentic reference samples, 120 laboratory-adulterated samples, and 18 commercial samples. The commercial samples were not used for model construction or optimization and were analyzed separately as an independent commercial screening set.

#### 2.3.2 Principal Component Analysis

Principal component analysis (PCA) was employed as an unsupervised exploratory method to examine the intrinsic distribution of authentic and laboratory-adulterated guaraná samples and the progressive spectral variation associated with increasing synthetic caffeine addition levels. The PCA model was constructed using the preprocessed LF ^1^H NMR spectral matrix described in Section 2.3.1. The matrix comprised 160 samples, including 40 authentic reference samples and 120 laboratory-adulterated samples.

The spectra used for PCA were normalized using the norm 1 and mean-centered before model construction. No additional variance scaling was applied. PCA was performed using the γ-GUI graphical interface, implemented in MATLAB 2023a. Principal components were calculated by singular value decomposition (SVD), according to **X = TP** ^**T**^ **+ E**, where **X** represents the spectral data matrix, **T** the score matrix, **P** the loading matrix, and **E** the residual matrix. Because PCA is an exploratory unsupervised method, model quality and interpretability were assessed based on the cumulative explained variance, the consistency of the sample distribution in the score space, and the chemical interpretability of the loading profiles. The first three principal components were retained for graphical representation and interpretation.^32,33^

#### 2.3.3 DD-SIMCA Authentication Model

For the authentication and qualitative classification of guaraná, the DD-SIMCA algorithm was implemented in Python, using customized routines based on the approach proposed by Zontov et al.^29^ Unlike conventional discriminant methods, DD-SIMCA is a one-class classification approach that focuses exclusively on modeling the intrinsic characteristics of the authentic target class. In the present study, the target-class model was constructed using the 40 authentic Maués guaraná samples, whereas the 120 laboratory-adulterated samples were used as the nontarget class to assess the model’s ability to reject samples that were inconsistent with the authentic spectral profile. The 18 commercial samples were not used for model construction, optimization, or calculation of classification-performance metrics; instead, they were subsequently projected into the optimized DD-SIMCA decision space as an independent external validation set.

The target-class samples were partitioned using the Kennard–Stone algorithm, with 67% used for calibration (*n* = 27) and the remaining 33% for prediction (*n* = 13). The model was constructed by PCA decomposition of the target calibration matrix. Samples deviating from the modeled profile were classified as noncompliant or anomalous relative to the authentic Maués guaraná class, rather than automatically identified as adulterated, because rejection by a one-class model does not confirm a specific type of fraud.^26,34^ The classification process is based on the calculation of the leverage distance (Score Distance, *h*) and the orthogonal residual distance (*v*), whose acceptance limits are defined by empirical degrees of freedom (*N*_*h*_ e *N*_*v*_) fitted to a Chi-square (χ^2^) distribution. Using the “Compliant” strategy, the significance level (α) was iteratively adjusted to optimize the decision boundary and minimize the global error.^26,30^

The robustness and predictive capacity of the developed models were rigorously evaluated through specific statistical parameters for each chemometric approach. For the authentication models, leave-one-out cross-validation (LOOCV) was employed as a strategy to assess performance without sampling bias. The fundamental metrics calculated included accuracy (ACC), Matthews correlation coefficient (MCC), sensitivity (SEN), specificity (SPE), and overall efficiency (EFI). These metrics were derived from the values of true positives (TP), true negatives (TN), false positives (FP), and false negatives (FN), providing a comprehensive assessment of the reliability and accuracy of the model’s classifications (eqs 1–5).

1 $$\mathrm{SEN}=\frac{\mathrm{TP}}{\mathrm{TP}+\mathrm{FN}}$$

2 $$\mathrm{SPE}=\frac{\mathrm{TN}}{\mathrm{TN}+\mathrm{FP}}$$

3 $$EFI=\sqrt{SEN\times SPE}$$

4 $$\mathrm{ACC}=\frac{\mathrm{TN}+\mathrm{TP}}{\mathrm{TN}+\mathrm{TP}+\mathrm{FN}+\mathrm{FP}}$$

5 $$\mathrm{MCC}=\frac{\left(\mathrm{TP}\times \mathrm{TN}\right)-\left(\mathrm{FP}\times \mathrm{FN}\right)}{\sqrt{\left(\mathrm{TP}+\mathrm{FP}\right)\left(\mathrm{TP}+\mathrm{FN}\right)\left(\mathrm{TN}+\mathrm{FP}\right)\left(\mathrm{TN}+\mathrm{FN}\right)}}$$

The selection of the optimal DD-SIMCA model was primarily based on maximizing the EFI, ensuring a balance between the correct classification of authentic samples and the rejection of anomalous ones. Given the inherent class imbalance in the data set, ACC becomes susceptible to majority-class bias; therefore, it was not utilized for model selection, being reported merely as an auxiliary descriptor. To corroborate the robustness of the models selected via EFI and specifically account for the unequal class sizes, the MCC was integrated into the evaluation process. The MCC served as a critical supporting metric because it mathematically accounts for all classification outcomes (true/false positives and negatives), ensuring a reliable and unbiased performance assessment even in the presence of imbalanced data.^35^ To mitigate the risk of overfitting, the number of PCs was optimized by analyzing metric performance relative to model complexity, complemented by diagnostic tools such as the extreme plot (Figure S1 in Supporting Information (SI)). Model stability was tested under different significance levels (α = 0.01 and 0.05) and validated with an external data set to confirm its generalizability on samples not used during calibration.

To elucidate the chemical basis of the classification and identify the spectral variables (NMR chemical shifts) responsible for guaraná authentication, the Discriminatory Power (DP) metric was applied in conjunction with the DD-SIMCA model. DP is a diagnostic chemometric tool that quantifies the individual capacity of each variable to differentiate the authentic class from adulterated samples.^35^ In this approach, the DP was calculated based on the variance of the orthogonal residuals generated by projecting the samples onto the PCA model of the target class. Mathematically, the metric was obtained through the square root of the ratio between the residual variance of the adulterated (nontarget) samples and the residual variance of the pure guaraná (target) samples. Consequently, variables presenting high DP values represent spectral regions that were significantly disturbed by fraud and are not well described by the authentic guaraná subspace, standing out as the primary chemical markers responsible for evidencing adulteration within the matrix.^36,37^

#### 2.3.4 Quantification of Synthetic Caffeine Adulteration Levels Using PLSR

Partial least-squares regression (PLSR) was employed to establish a multivariate calibration relationship between preprocessed LF ^1^H NMR spectra and known levels of synthetic caffeine addition. The data set for the PLSR comprised 160 laboratory-prepared samples, including 40 authentic guaraná samples, assigned a response value of 0%, and 120 laboratory-adulterated samples, with synthetic caffeine addition levels ranging from 1 to 10% (w/w). The predictor matrix, **X**, contained the intensities of the 6.891 spectral variables retained after the preprocessing procedures described in Section 2.3.1, while the response vector, **y**, contained the corresponding level of synthetic caffeine addition for each sample. The 18 commercial samples were not used for model construction, as their actual synthetic caffeine contents were unknown; they were subsequently evaluated as an external validation set.

To ensure statistically representative calibration and test subsets, the 160 laboratory-prepared samples were partitioned into 67% for calibration and 33% for held-out testing using the Sample Set Partitioning Based on Joint X–Y Distances (SPXY) algorithm.^25^ This resulted in 107 samples in the calibration subset and 53 samples in the test subset. SPXY considers the joint distances between samples in both the spectral and response spaces, thereby preserving the variability of *X* and *y* in both subsets. The decomposition of the predictor and response matrices into latent scores and loadings was performed using the Statistically Inspired Modification of Partial Least Squares (SIMPLS) algorithm.^38^ Model complexity was optimized by applying leave-one-out cross-validation (LOOCV) exclusively to the calibration subset. The number of latent variables (LVs) was selected based on the minimum root-mean-square error of cross-validation (RMSECV), while also considering model parsimony and stabilization of the cross-validation error. The final PLSR model retained six LVs.

Model performance and predictive reliability were evaluated using the coefficient of determination for calibration (*R*_cal_^2^), root-mean-square error of calibration (RMSEC), coefficient of determination for prediction (*R*_pred_^2^), and root-mean-square error of prediction (RMSEP).^39,40^ Model robustness was assessed from the agreement between calibration and held-out test performance, the similarity between RMSEC and RMSEP, and the absence of substantial systematic deviations between the reference and predicted values. Analytical figures of merit were additionally calculated to characterize the sensitivity of the multivariate calibration models. These included the standard error of the method (SEM), inverse analytical sensitivity (IAS), and minimum and maximum limits of detection and quantification (LOD_min/max_ and LOQ_min/max_). These parameters were estimated according to IUPAC-consistent recommendations for multivariate calibration.^41,42^ Variable importance in projection (VIP) scores were also calculated to identify the spectral variables contributing most strongly to the prediction of synthetic caffeine addition levels.

## 3 Results and Discussion

### 3.1 LF ^1^H NMR Chemical Profiles

The analysis of the LF ^1^H NMR spectra, presented in Figure 2, provides a comprehensive overview of the metabolic profile of powdered guaraná and highlights the spectral changes associated with synthetic caffeine addition. Because the simultaneous overlay of all individual spectra would substantially reduce visual clarity, Figure 2 presents the mean spectral profiles of the investigated groups. To provide a complete representation of within-class spectral variability, the individual spectra are presented in Figure S2 in the SI. It is essential to consider that Low-Field (LF) NMR has inherent spectral resolution limitations compared to high-field equipment. These limitations can hinder the precise identification of minor metabolites due to signal overlap, particularly in the polyphenol and sugar regions.^19,24^ Such restrictions stem from the lower magnetic field strength (1.5 T), which reduces chemical-shift dispersion and analytical sensitivity. To mitigate these effects and ensure data integrity for the subsequent chemometric stage, rigorous digital processing protocols were applied, including baseline adjustments and phase corrections. The assignment of chemical signals was consolidated by consulting the Human Metabolome Database (HMDB) and specific literature on *P. cupana*.^2,27^ This ensured reliability in identifying authenticity markers, such as the characteristic caffeine singlets and signals from other compounds like catechins, fatty acids, and amino acids.^21,24^

**Figure 2:**
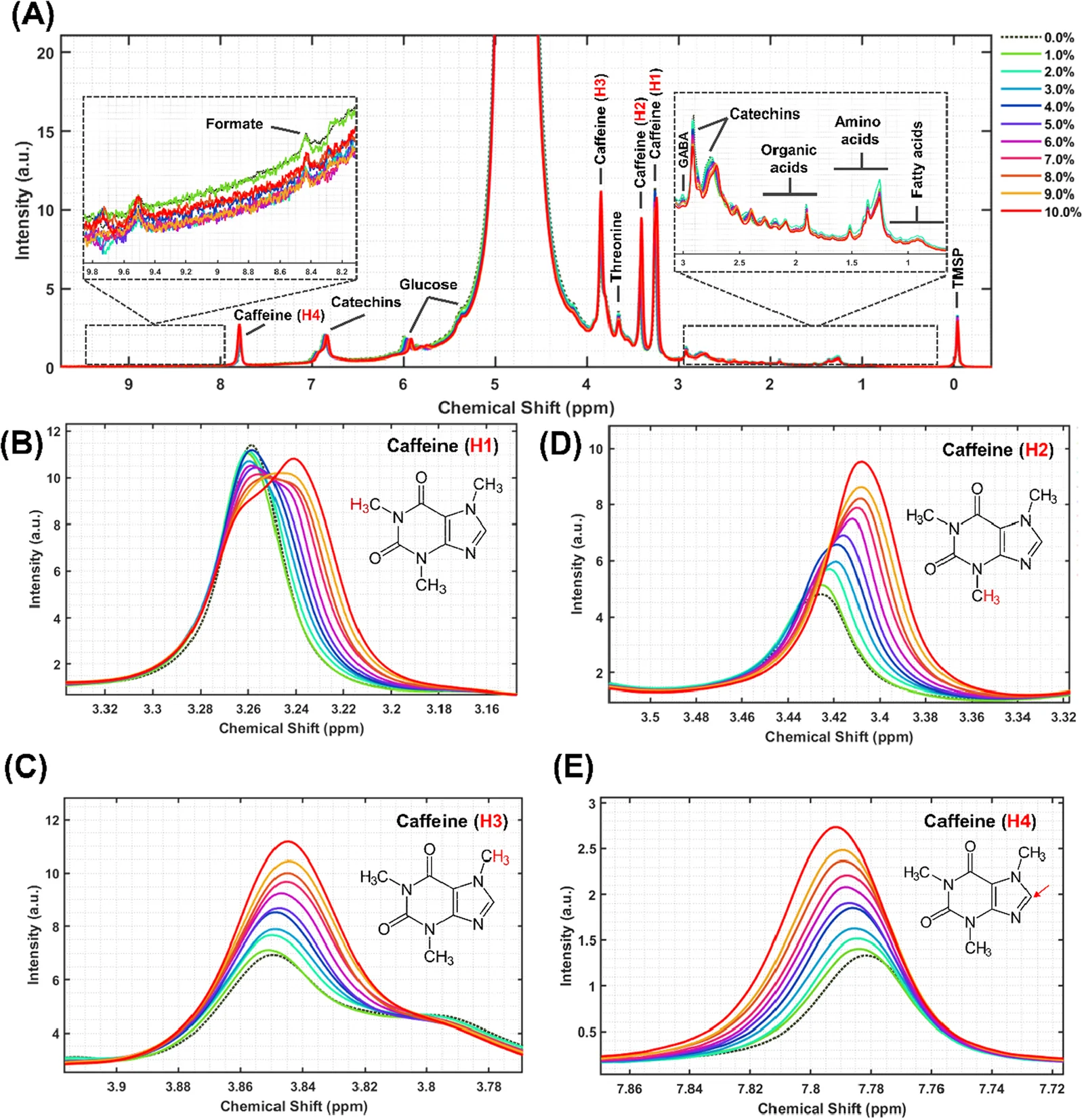
LF ^1^H NMR spectra of powdered guaraná samples with increasing levels of synthetic caffeine adulteration (0–10%). (A) Full spectrum (δ 0–10 ppm). (B–E) Enlarged views of the caffeine resonances: (B) H1 (1-CH_3_) and (D) H2 (3-CH_3_) in the *N*-methyl region (δ 3.0–4.0 ppm), (C) H3 (7-CH_3_), and (E) H4 (H-8) in the aromatic region (δ ∼ 7.7–8.0 ppm).

Figure 2A presents the overlaid spectra of authentic guaraná samples and those adulterated with synthetic caffeine (0–10%), showing that while the most abundant metabolic signals of the matrix (such as catechins, sugars, and amino acids) remain preserved, there is a progressive and proportional increase in the intensity of the caffeine peaks. The magnifications of the caffeine peaks shown in Figure 2B–E, in which the labels H1–H4 were adopted to facilitate visual identification of the corresponding protons and their spectral signals, reveal a site-specific response to increasing synthetic caffeine concentration. The *N*-methyl resonances H1 (1-CH_3_), H2 (3-CH_3_), and, to a lesser extent, H3 (7-CH_3_), together with signals from matrix constituents such as catechins, exhibited progressive upfield shifts toward lower δ values. This increased shielding may be associated with concentration-dependent self-association of caffeine molecules in aqueous solution and heteroassociation with endogenous guaraná constituents, particularly polyphenols, through π–π stacking and related intermolecular interactions. Such molecular arrangements generate anisotropic magnetic environments that can increase shielding around the *N*-methyl groups as molecular density increases.^43,50^

In contrast, the aromatic H4 resonance (H-8; δ 7.7–8.0 ppm) showed a slight but consistent downfield displacement toward higher δ values. The opposite behavior of H4 likely reflects its distinct local electronic environment within the heteroaromatic xanthine ring, where the geometry of caffeine–caffeine and caffeine–matrix associations may produce relative deshielding rather than the increased shielding observed for the *N*-methyl groups.^43,45^ Therefore, the concentration-dependent chemical-shift response appears to be proton-site specific and may result from the combined effects of molecular self-association, matrix interactions, and the different anisotropic environments experienced by the *N*-methyl and aromatic protons. Despite this small downfield displacement, the H4 signal exhibited a comparatively regular concentration-dependent intensity response, supporting its relevance as a robust variable for chemometric quantification.^2^

In contrast, the other markers remain consistent, reflecting the preserved metabolic profile of the guaraná seed. In the aromatic region (δ ∼ 7.0 ppm), signals attributed to phenolic compounds such as catechins stand out, while the presence of glucose is noted in the δ ∼ 6.0 ppm region. The aliphatic region (inset from δ 1.0 to 3.0 ppm) reveals a complex mixture of fundamental metabolites preserved in the powder, including GABA, organic acids, amino acids, and fatty acids.^27^ Additionally, in the region between δ 8.0 and 9.5 ppm, there is a formate peak whose signal also exhibits concentration dependence. These results indicate that the primary metabolic profile of guaraná supports the addition of caffeine without losing its fundamental matrix characteristics; however, the differences evidenced by the intensities and shifts identified in the LF ^1^H NMR spectra show great potential for traceability and authentication of authentic samples, as well as indicating the level of adulteration with synthetic caffeine.

### 3.2 Exploratory Analysis

Prior to multivariate modeling, the evaluated normalization strategies were systematically compared to determine the preprocessing conditions that provided the most stable and interpretable chemometric results. Among the tested approaches, norm 1 consistently provided the most favorable performance across the exploratory, classification, and regression analyses. This normalization reduced variations associated with the overall spectral intensity and minor differences in extraction efficiency while preserving chemically meaningful relative signal patterns. Therefore, norm 1 was selected for subsequent PCA, DD-SIMCA, and PLSR analyses.

The PCA analysis presented in Figure 3 revealed a general tendency toward separation between authentic and laboratory-adulterated guaraná powder samples, particularly along PC1. However, partial overlap was observed between the authentic group and some samples containing 1–2% added synthetic caffeine, indicating that PCA alone is insufficient for definitive authentication at low adulteration levels. Accordingly, PCA was used as an exploratory approach to visualize the main sources of spectral variability, whereas formal authentication was performed using the DD-SIMCA one-class model. The first three principal components explained 86.20% of the total variance, with PC1 accounting for 61.33%, PC2 for 16.32%, and PC3 for 8.55%, providing an informative three-dimensional representation of the data set. The main concentration-dependent trend occurred along PC1, where authentic samples were predominantly distributed in the positive-score region, whereas samples with increasing levels of synthetic caffeine addition generally shifted toward more negative scores. This trend became more pronounced at higher adulteration levels, while samples containing 1–2% added caffeine remained closer to, or partially overlapped with, the authentic group.

**Figure 3:**
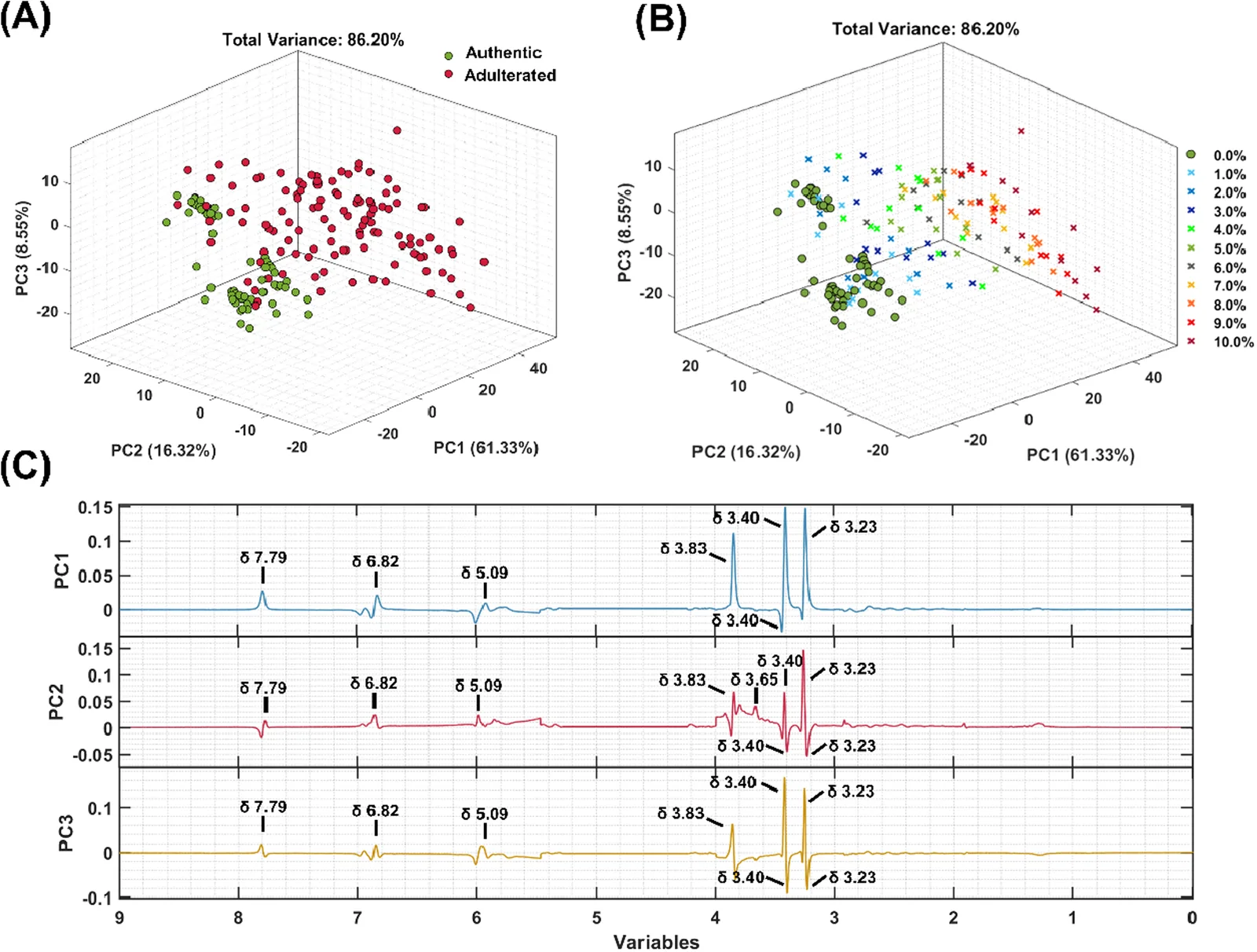
Principal component analysis (PCA) results based on the LF ^1^H NMR spectra of the guaraná samples. (A) Three-dimensional score plot of the authentic samples (green) and those adulterated with synthetic caffeine (red). (B) Score plot with samples color-coded according to the level of synthetic caffeine addition (0–10%). (C) Loading plots corresponding to principal components PC1, PC2, and PC3.

The coding by caffeine addition level reveals that PC1 functions as a true quantitative axis, samples with 7–10% adulteration exhibit the most negative scores, whereas those with low levels (1–3%) tend to approach the authentic group, highlighting the method’s sensitivity in detecting initial stages of fraud. The chemical significance of this behavior is directly elucidated by the PC1 loadings, which show the highest contributions in the regions of δ 3.23, δ 3.40, δ 3.83, and δ 7.79 ppm. These chemical shifts correspond to the three methyl groups (H1–3) and the aromatic proton (H-4) of the xanthine ring,^27^ confirming that the samples’ behavior in the first component is governed by the addition of synthetic caffeine into the original matrix.

In addition to the global variation caused by adulteration, the PCA indicated substructure within the authentic-sample cluster. To investigate this natural variability independently of the effect of synthetic caffeine addition, an additional PCA was performed using only the 40 authentic, nonadulterated samples (Figure S3). Samples from Farm 1 formed a more clearly defined group, whereas samples from Farms 2 and 3 showed grouping tendencies with partial overlap. This distribution may be associated with the greater geographical separation of Farm 1, while Farms 2 and 3 are geographically closer and managed by the same producer, potentially sharing similar cultivation and postharvest practices. The PC2 loadings showed relevant contributions in the δ 3.1–4.0 ppm region, mainly associated with threonine and oxygenated protons from carbohydrates and sugars. Additional contributions were observed in the aromatic region, approximately between δ 5.7 and 7.1 ppm, where signals from native polyphenols, including catechins and epicatechins, are expected.^2,27,46^ These findings suggest that PC2 captures natural matrix variability associated with edaphoclimatic conditions and production practices, consistent with studies showing that terroir- and management-related markers may be expressed in secondary principal components.^47,48^

PC3 contributed to the three-dimensional dispersion of the samples and captured subtle spectral variations that were not fully described by PC1 and PC2. Notably, the PC3 loading profile showed sign-changing contributions around δ 3.23, 3.40, 3.83, and 7.79 ppm, corresponding to the H1, H2, H3, and H4 caffeine resonances shown in Figure 2B–E. The presence of positive and negative loading features at adjacent spectral positions is consistent with the redistribution of signal intensity produced by small resonance shifts. Accordingly, PC3 appears to capture, at least in part, the progressive upfield displacement of H1–H3 and the slight downfield displacement of H4 observed as the synthetic caffeine concentration increased.^43,45^ This behavior was most pronounced for the H1 and H2 signals and less intense for H3 and H4, in agreement with the magnitude of the shifts observed in the enlarged spectra. Additional oscillatory contributions around δ 5.09 and 6.82 ppm suggest that PC3 also reflects subtle perturbations of endogenous matrix constituents, potentially associated with caffeine–polyphenol interactions and concentration-dependent changes in the spectral environment.^49,50^

### 3.3 Guaraná Authentication

For the authentication of the guaraná samples, the DD-SIMCA approach was applied to the LF ^1^H NMR spectra, which were processed and normalized using mean centering. Unlike purely discriminatory methods, DD-SIMCA is highly recommended for authentication tasks because it focuses on rigorously modeling the boundaries of the target class. In this study, the target class was defined exclusively by the authentic guaraná samples, while the samples adulterated with synthetic caffeine were used as the external class. For regulatory and quality control purposes, the model was optimized to maximize SEN and SPE. The performance of the models as a function of the number of PCs evaluated is detailed in Figure S4 in the SI.

In regulatory inspection operations, the critical objective is to ensure that no authentic sample is falsely rejected while simultaneously guaranteeing the rejection of fraudulent products. To achieve this level of reliability, the algorithm optimization involved testing different significance values (α) and applying the compliant mode, which considers only target-class samples during the calibration stage to refine the model boundaries. As a result of this optimization, the model achieved high performance, reaching 100% sensitivity (with no false negatives recorded) during the calibration stage. The high reliability of the method is further corroborated by the elevated accuracy rates (100% in calibration and 99.28% in prediction), an efficiency of 99.58%, and a Matthews Correlation Coefficient (MCC) higher than 0.96.

However, it is crucial to clarify the precise scope of these model boundaries. Because the target class was constructed using authentic reference samples predominantly sourced from the Maués region, the DD-SIMCA model naturally established a highly specific chemical boundary based on this regional fingerprint. Consequently, the defined authenticity limits reflect “Maués guaraná” rather than a universal profile for all genuine guaraná. This geographical restriction explains why commercial samples from other regions, even if potentially unadulterated, were rejected by the model for falling outside this strict high-quality profile. Future efforts can systematically expand the target class by incorporating authentic reference samples from diverse geographical origins, thereby generalizing the model into a comprehensive screening tool for nationwide regulatory inspection and routine quality control. The decision space of this specialized model is visualized through the acceptance plots. To accommodate large deviations and clearly highlight the final acceptance boundary calculated at α = 0.01, both distances were logarithmically transformed into the forms log­(1 + OD/OD0) and log­(1 + SD/SD0).

Figure 4A illustrates the performance of the binary model (Authentic vs Globally Adulterated). It can be observed that the authentic samples (green) are entirely concentrated within the chi-square boundary defining the acceptance region, indicating an excellent modeling of the metabolic “fingerprint” of the matrix. In contrast, the adulterated samples (red) are predominantly positioned outside this boundary. This demonstrates that adulteration not only alters the global covariance structure of the spectrum but also introduces residual variations not explained by the target-class model. To assess the applicability of this binary model in a real regulatory inspection scenario, commercial market samples (MKT) were projected into this space. All MKT samples were classified outside the acceptance region, clustering close to the adulterated samples, indicating a strong suspicion of noncompliance among products available on the market and confirming the effectiveness of the method as a rapid regulatory screening tool. The individual LF ^1^H NMR spectra of the 18 commercial samples are presented in Figure S5. This representation enables direct visualization of the spectral variability and overall similarities between the two sample groups without assigning a confirmed adulteration status to the commercial samples.

**Figure 4:**
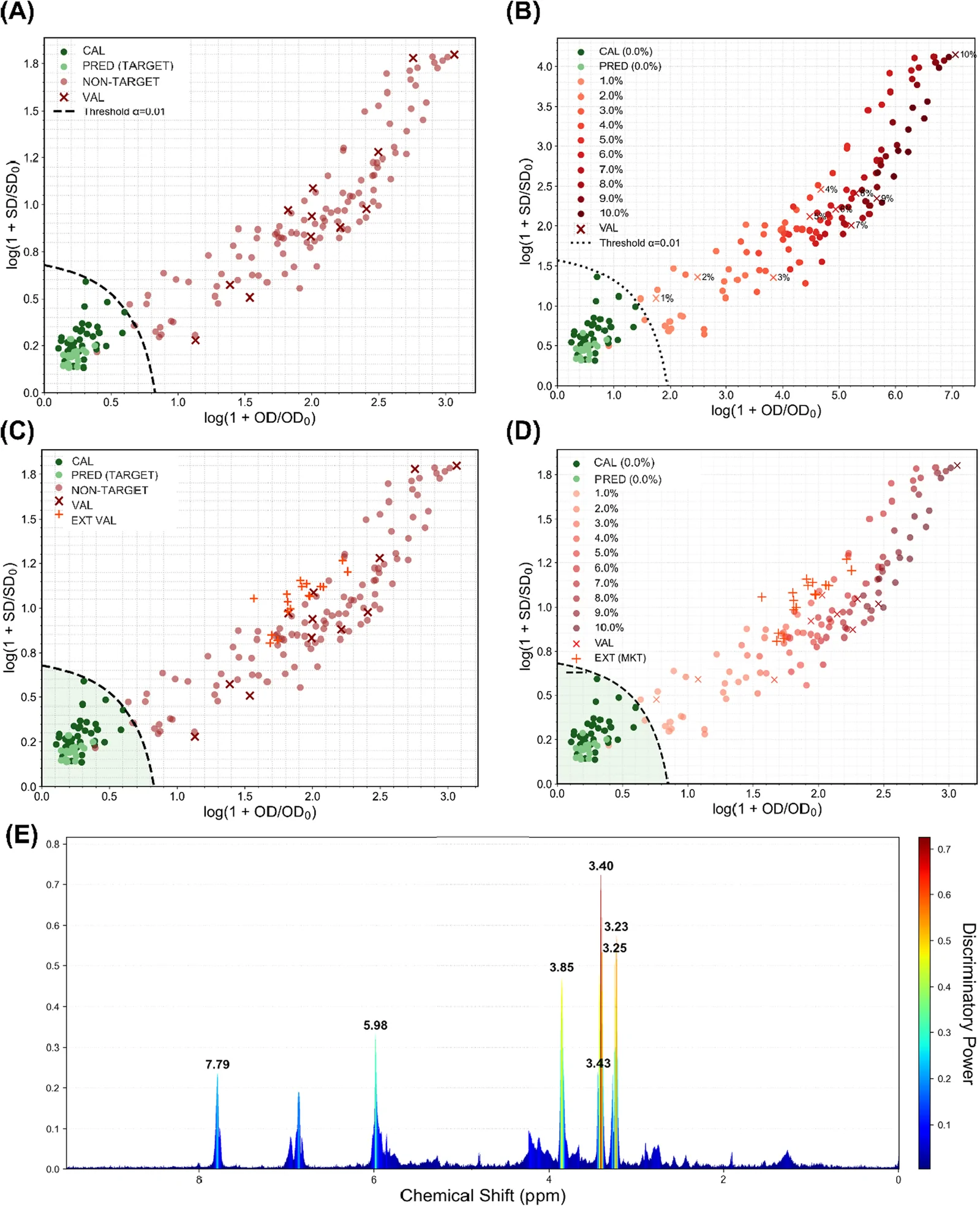
Results of the DD-SIMCA modeling based on LF ^1^H NMR spectra for guaraná powder authentication. (A) Acceptance plot of the binary model for the target (authentic, green) and external (adulterated, red) classes (α = 0.01). (B) Acceptance plot with adulterated samples color-coded by percentage levels of synthetic caffeine addition (1–10%). (C) Projection of external commercial market samples (MKT, “X”) into the binary model decision space. (D) Projection of MKT samples into the latent space representing adulteration levels. (E) Average ^1^H NMR spectrum overlaid with a variable discriminant power heatmap.

To further deepen the analytical capability, Figure 4B presents the external class according to synthetic caffeine addition levels (1.0–10.0%). A clear distancing gradient was observed, with low-level samples (∼1.0 to 3.0%) located near the acceptance boundary and higher-level samples (∼8.0 to 10.0%) projected farther from the authentic class. One sample containing 1% added caffeine remained within the acceptance region, representing a false acceptance of a nontarget sample. This likely reflects overlap between the small spectral perturbation caused by low-level adulteration and the natural variability of authentic guaraná. Because the boundary at α = 0.01 prioritizes sensitivity, borderline nontarget samples may occasionally be accepted.^29,34^ This result indicates a localized limitation at very low adulteration levels rather than a general model failure. Commercial samples were distributed across regions associated with different laboratory-spiked levels, but these positions should be interpreted only as spectral similarities, not as definitive evidence of specific adulteration percentages.

However, it is important to emphasize that the rejection of commercial samples by the DD-SIMCA model requires further investigation to unequivocally confirm fraud, as it does not exclusively indicate the addition of synthetic caffeine. Since the product labels did not specify the exact geographical origin of cultivation, the observed spectral divergence (Figure S6) may arise from terroir differences or from substantial variations in agro-industrial processing. From a chemometric perspective, DD-SIMCA constructs a rigorous acceptance hypervolume fitted exclusively to the variance of the target class (guaraná from Maués, Amazonas); therefore, external factors capable of altering secondary metabolites cause the model to fail in reconstructing the modified profile, drastically inflating the residual variance (orthogonal distance, qi). Consequently, the sample is projected outside the acceptance boundary, reflecting an anomaly relative to the calibration set, which demands complementary analyses to distinguish intentional adulteration from natural but unmodeled variability.^51,52^

The statistical interpretation is directly supported by the metabolic profile of the matrix, as illustrated in Figure 4E. The plot showing the overlaid average spectrum together with the discriminant power intensity for variable selection demonstrates that the most critical regions for class separation are not random artifacts. The regions with the highest discriminant power correspond precisely and consistently to the markers previously discussed: the aromatic region at δ ∼ 7.7–8.0 ppm (where the H-8 proton of caffeine is located, along with phenolic compounds) and the aliphatic region at δ ∼ 3.2–4.0 ppm (which encompasses the *N*-methyl groups of caffeine, corresponding to 1-CH_3_, 3-CH_3_, and 7-CH_3_, as well as carbohydrates).^2,27^ The high statistical weight of these regions confirms that the model rejects adulterated samples based on the dilution of characteristic guaraná compounds and the disproportionate increase in signals associated with the adulterant. This integration demonstrates that the constructed chemometric model possesses a fully interpretable chemical basis, acting as a mirror of the molecular perturbations caused by fraud.

### 3.4 Quantification of Guaraná Adulteration

The present approach did not employ absolute qNMR based on the integration ratio between caffeine and an internal quantitative standard. Instead, synthetic caffeine addition was estimated through multivariate calibration, in which the normalized LF ^1^H NMR spectral matrix was related to the known addition levels used during model development. The chemometric optimization of the model and the selection of the number of LVs are illustrated in Figure 5A. The cumulative variance analysis showed that approximately 99% of the information associated with the response variable *Y*, corresponding to the added synthetic caffeine concentration, was explained by only three to four LVs. Correspondingly, the RMSECV decreased sharply and reached a plateau at approximately five LVs, while the *R* ^2^ approached 1.0. This relatively simple latent structure indicates a strong and predominantly linear relationship between the processed 1H NMR spectral information and the concentration of added synthetic caffeine. Although the inclusion of additional LVs provided only marginal mathematical gains, the small difference between the errors of the final model, with an RMSEC of 0.28% and an RMSEP of 0.36%, indicates no substantial overfitting and supports the model’s predictive robustness for held-out laboratory-spiked samples within the investigated calibration range.

**Figure 5:**
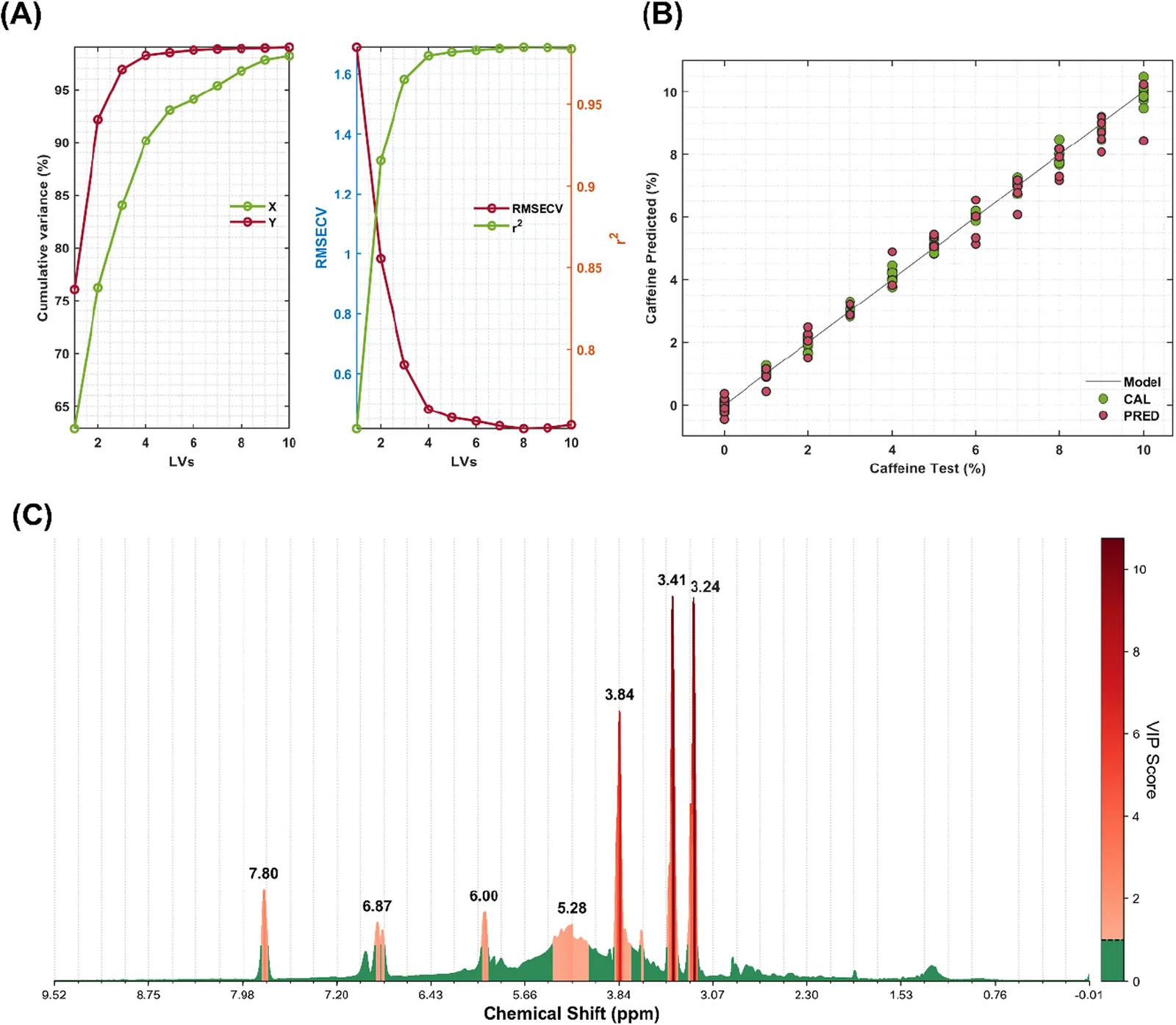
Multivariate calibration by PLSR for the quantification of synthetic caffeine in guaraná powder. (A) Model optimization plot showing the cumulative variance explained for *X* and *Y* matrices, RMSECV, and *R* ^2^ for the evaluation of Latent Variables (LVs). (B) Correlation plot between predicted and actual caffeine concentrations (%) for the calibration (CAL, green) and external prediction (PRED, red) sets. (C) Variable Importance in Projection (VIP) scores superimposed on the ^1^H NMR spectral profile.

The predictive performance of the model is further illustrated by the correlation plot between the reference and predicted concentrations shown in Figure 5B. Both the calibration (CAL) and external prediction (PRED) sample sets were distributed with minimal dispersion and close to the ideal 1:1 line. The model achieved *R*_cal_^2^ = 0.9931 and *R*_pred_^2^ = 0.9891, indicating strong agreement between the reference and predicted values and no evident systematic bias. No marked curvature or response saturation was observed at the upper end of the investigated range, approximately 10%, while a consistent predictive response was maintained at the lowest experimentally evaluated addition level.

The analytical figures of merit further supported the sensitivity of the method, with an analytical sensitivity of 42.50, limits of detection ranging from 0.08 to 0.10%, and limits of quantification ranging from 0.24 to 0.30%. These model-derived parameters indicate that the spectral information captured by the PLSR model is highly sensitive to low-level variations associated with synthetic caffeine addition. This analytical capability enhances the applicability of the proposed workflow as a first-line screening strategy for identifying subtle alterations in the guaraná matrix. Such sensitivity is relevant to food labeling and consumer safety because regulatory authorities, including ANVISA, EFSA, and FDA, establish recommendations and safety limits for caffeine consumption in foods and dietary supplements.^9–11^ In this context, the proposed method may support label-compliance assessment and the prioritization of potentially noncompliant samples for subsequent confirmatory analysis.

The chemical significance of the regression was evaluated using Variable Importance in Projection (VIP) scores, as illustrated in Figure 5C. Although the complete spectra presented in Figure 2 retain the intense water resonance for visualization purposes, the corresponding δ 4.50–5.00 ppm region was digitally excluded from the spectral data matrix before normalization and chemometric modeling, as described in the spectral preprocessing section. Phase and baseline corrections were also applied before multivariate analysis to minimize peak-shape distortions and instrumental baseline variations. Consequently, the principal water peak did not directly contribute to the construction of the PLSR model.

The variables with the highest VIP scores corresponded to the characteristic caffeine resonances at approximately δ 3.24, 3.41, 3.84, and 7.80 ppm, assigned to the three *N*-methyl groups and the C8–H proton, respectively. Because these high-VIP variables were located outside the excluded water region, their correspondence with structurally meaningful caffeine signals provides strong evidence that the latent information used by the model was primarily driven by caffeine-related spectral variations. Secondary VIP contributions were observed at approximately δ 5.28, 6.00, and 6.87 ppm and may reflect changes in endogenous guaraná constituents, including oxygenated and phenolic compounds, associated with the progressive addition of synthetic caffeine and the consequent relative dilution of the original matrix. Thus, the full-spectrum PLSR approach incorporated both direct caffeine resonances and matrix-related spectral perturbations rather than relying exclusively on a single analytical peak.

Nevertheless, the intense water resonance remains a methodological consideration in aqueous low-field 1H NMR. Although spectral cropping prevented the principal water peak from contributing directly to the PLSR model, minor effects associated with broad water-signal tails, local baseline variations, and spectral overlap near the excluded interval cannot be completely ruled out because of the limited resolution of the 60 MHz instrument. For this reason, the secondary contribution observed near δ 5.28 ppm should be interpreted cautiously. However, the predominance of characteristic caffeine resonances among the highest VIP scores, together with the satisfactory predictive performance obtained for the held-out laboratory-spiked samples, supports that the model was primarily based on chemically meaningful caffeine-related information rather than on the water signal.

Similarly, because the extracts were analyzed under native, nonbuffered conditions, minor pH-dependent variations in the chemical shifts of ionizable matrix constituents cannot be completely excluded. Nevertheless, the variables with the highest VIP scores corresponded predominantly to the characteristic caffeine resonances at approximately δ 3.24, 3.41, 3.84, and 7.80 ppm. Together with the strong agreement between reference and predicted concentrations for the held-out laboratory-spiked samples, this finding indicates that caffeine-related spectral variation remained the dominant quantitative information under the preparation conditions employed. These results support the robustness of the PLSR model within the native pH variability represented by the investigated sample set. However, because extract pH was not independently measured or systematically varied, robustness against deliberately controlled pH changes was not specifically assessed.

To assess the prospective applicability of the method under real-market inspection conditions, the optimized PLSR model was applied to 18 commercial samples (MKT1–MKT18). Because the true synthetic caffeine contents and adulteration status of these products were unknown, the commercial samples were treated as an external validation set. Most samples generated synthetic caffeine-equivalent estimates close to zero. Several estimates were below or near the model’s LOQ of 0.239–0.305%, while negative predictions, arising from statistical variation around the lower boundary of the calibration model, were reported as 0.0% for practical interpretation. Three commercial samples generated estimates above 1%: MKT7, MKT8, and MKT13, with predicted values of 1.801, 1.549, and 1.030%, respectively (Table 1).

**Table 1:** Statistical Performance Parameters, Analytical Figures of Merit (FoM), and Results of External Validation of the PLSR Model Developed for the Quantification of Caffeine Adulteration in Guaraná Samples

statistical parameters of PLSR model				
LVs	*R* ^2^ (CAL)	RMSEC	*R* ^2^ (PRED)	RMSEP
6	0.9931	0.2841	0.9891	0.3636

figures of analytical merit (FoM)				
SEM	LOD (min)	LOD (max)	LOQ (min)	LOQ (max)
1.359	0.078	0.101	0.239	0.305

external validation		
sample	expected alteration (%)	adjusted adulteration (%)
MKT1	0.177	0.177
MKT2	0.444	0.444
MKT3	–0.169	0.000
MKT4	–0.082	0.000
MKT5	0.397	0.397
MKT6	0.417	0.417
MKT7	1.801	1.801
MKT8	1.549	1.549
MKT9	0.717	0.717
MKT10	0.790	0.790
MKT11	–0.713	0.000
MKT12	–0.328	0.000
MKT13	1.030	1.030
MKT14	0.557	0.557
MKT15	–0.087	0.000
MKT16	0.139	0.139
MKT17	–0.325	0.000
MKT18	–0.444	0.000

These results indicate that the spectral profiles of these samples were consistent with those observed for laboratory-spiked samples containing low levels of synthetic caffeine. However, because no independent confirmatory analytical technique was employed and the true adulteration status of the commercial samples was unknown, the predicted values must be interpreted as model-based, presumptive synthetic caffeine-equivalent estimates rather than definitive evidence of adulteration. Although the samples were projected within regions of the PLSR calibration space associated with low levels of synthetic caffeine addition, this finding does not unequivocally demonstrate that synthetic caffeine was present in the commercial products. Confirmation of the presence and origin of additional caffeine would require an independent analytical method capable of distinguishing natural from synthetic sources, such as stable isotope ratio analysis. Therefore, the proposed PLSR model should be regarded as a sensitive first-line screening tool for identifying commercial samples that warrant a subsequent confirmatory investigation.

The combined DD-SIMCA and PLSR results provide complementary information regarding the commercial samples. Although DD-SIMCA identified these samples as anomalous relative to the authentic Maués guaraná class, the PLSR model generated low or near-zero synthetic caffeine-equivalent estimates for most of them. This pattern suggests that many of the deviations identified by DD-SIMCA may reflect natural or processing-related variability rather than spectral changes consistent with synthetic caffeine addition. Factors such as geographical origin outside Maués, differences in agricultural practices, and variations in roasting time and temperature may alter the natural balance of polyphenols, carbohydrates, and other metabolites, thereby increasing the distance of commercial samples from the DD-SIMCA target class.^51,52^

Nevertheless, in the absence of independent confirmatory measurements, the PLSR predictions cannot conclusively establish either the presence or absence of synthetic caffeine in the commercial products. Therefore, the complementary application of DD-SIMCA and PLSR provides a hierarchical screening strategy: DD-SIMCA identifies deviations from the authentic reference fingerprint, whereas PLSR estimates whether these deviations are consistent with the spectral pattern generated by controlled synthetic caffeine addition. This combined interpretation reduces the risk of attributing every anomalous commercial profile to confirmed adulteration and enables selected samples to be prioritized for a subsequent confirmatory analysis.

The ability to accurately confirm and quantify this specific type of fraud is not merely an analytical achievement, but a critical economic necessity. The urgency for robust authentication methods is fundamentally driven by the substantial financial incentive behind economically motivated adulteration. In the wholesale market, the average price of authentic, GI Maués guaraná powder fluctuates between $48.00 and $114.00 USD per kilogram due to its traditional and sustainable production chain. Conversely, industrial-grade synthetic anhydrous caffeine is readily available for approximately $12.00 to $20.00 USD per kilogram. Consequently, adulterating a single ton of guaraná powder with just 10% synthetic caffeine yields an illicit profit margin, or equivalent supply chain loss, exceeding $6000.00 USD. When weighed against these severe economic losses, the proposed LF ^1^H NMR methodology emerges as highly cost-effective. By completely eliminating the need for expensive high-field cryogenic maintenance and high-cost deuterated solvents, the operational cost per sample is reduced to mere fractions of a dollar. Ultimately, translating the spectral data into precise quantification allows industries and regulatory bodies to use these predictive models for direct trade arbitration, enabling them to establish fair-market price deductions or outright reject compromised batches.

## 4 Conclusions

This study demonstrated the potential of LF ^1^H NMR spectroscopy at 60 MHz combined with chemometric modeling for the authentication and quality control of powdered guaraná. PCA showed that synthetic caffeine addition was the main source of spectral variance, while secondary components captured natural metabolic differences associated with geographical origin and artisanal processing practices in Maués, Amazonas, Brazil.

The one-class DD-SIMCA model established rigorous statistical boundaries for authentic Maués guaraná, achieving 100% sensitivity and accuracy above 99%. Complementarily, the PLSR model showed strong predictive performance for laboratory-spiked samples, with *R*_pred_^2^ = 0.9891, RMSEP = 0.36%, and low model-derived limits of detection and quantification. When applied to commercial samples, the PLSR model generated presumptive synthetic caffeine-equivalent estimates that should be interpreted as screening results rather than definitive confirmation of adulteration.

The proposed workflow reduces dependence on deuterated and organic solvents and avoids chromatographic separation and cryogenic instrumentation, supporting its alignment with Green Chemistry principles. Nevertheless, because guaraná powder is a heterogeneous and particulate matrix, standardized aqueous extraction, homogenization, ultrasound-assisted extraction, centrifugation, and supernatant transfer are required before spectral acquisition. These additional operations increase the overall preparation time and represent a limitation compared with direct or preparation-free quantitative NMR methods. Therefore, the workflow should be regarded as comparatively streamlined and solvent-efficient rather than preparation-free.

Overall, the combined application of DD-SIMCA and PLSR demonstrated potential as a first-line screening strategy for identifying deviations from the authentic Maués guaraná fingerprint and prioritizing potentially noncompliant samples for confirmatory analysis. However, the current calibration domain is geographically restricted to authentic samples from Maués. Therefore, broader regulatory or forensic implementation will require expansion of the reference database to include samples from additional geographical origins, harvest periods, cultivars, and processing conditions, together with independent and interlaboratory validation. Within these limitations, the proposed platform may support the future development of more comprehensive authentication systems for guaraná and other complex food matrices.

## Supplementary Material



## Notes

Analytical greenness report; DD-SIMCA diagnostic and performance plots; LF ^1^H NMR spectral profiles of authentic, laboratory-adulterated, and commercial guaraná samples; PCA score plot; comparison of authentic and commercial guaraná samples; and gravimetric preparation of authentic and synthetic caffeine-adulterated samples (PDF)
