# Supplementary material for: Novel Analytical Workflow for Tracing Synthetic Caffeine Adulteration in Amazonian Guaraná (Paullinia cupana) Using Low-Field 1H NMR Fingerprinting and Chemometrics
Source: J Agric Food Chem. 2026 Aug 10;74(32):25744–59. doi: 10.1021/acs.jafc.6c08341 (PMC13495765; doi:10.1021/acs.jafc.6c08341)
Supplement: Supplementary file 1 [file jf6c08341_si_001.pdf]

Supporting Information

A novel analytical workflow for tracing synthetic caffeine adulteration in Amazonian guaraná (*Paullinia cupana*) using low-field ^1H NMR fingerprinting and chemometrics

Jelmir Craveiro de Andrade[a,b*], Pedro Lucas Caetano de Oliveira[a,b], Maxsueli Machado[a,b,c], Adriano Teixeira de Oliveira[a,b,d,e,f,g,h], Adriene Núzia de Almeida Santos[d,i], Carlos Adam Conte-Junior[a,b,d*]

[a]Center for Food Analysis (NAL), Technological Development Support Laboratory (LADETEC), Federal University of Rio de Janeiro (UFRJ), Cidade Universitária, Rio de Janeiro, RJ, 21941-598, Brazil.
[b]Laboratory of Advanced Analysis in Biochemistry and Molecular Biology (LAABBM), Department of Biochemistry, Federal University of Rio de Janeiro (UFRJ), Cidade Universitária, Rio de Janeiro, RJ, 21941-909, Brazil.
[c]Federal University of Mato Grosso (UFMT), Cuiabá 78060-900, MT, Brazil
[d]Center for Studies of Invertebrates and Vertebrates of the Amazon (NEIVA), Federal Institute of Education, Science and Technology of Amazonas (IFAM), Manaus Centro Campus (CMC), Manaus 69020-120, AM, Brazil
[e] Graduate Program in Animal Science and Fisheries Resources (PPGCARP), Faculty of Agricultural Sciences (FCA), Federal University of Amazonas (UFAM), University Campus, Manaus 69077-000, AM, Brazil
[f] Graduate Program in Veterinary Sciences (PPGCVET), Federal Institute of Education, Science and Technology of Amazonas (IFAM) and Nilton Lins University Center (CUNL), Manaus 69020-120, AM, Brazil
[g] Graduate Program in Biodiversity and Science Education in the Amazon (PPGBEC), Federal Institute of Education, Science and Technology of Amazonas (IFAM) and Amazonas State University (UEA), Manaus 69020-120, AM, Brazil
[h] Amazonas State University, Manau 69050-010, AM, Brazil
[i] Nilton Lins University Center (CUNL), Manaus 69058-020, AM, Brazil

*Corresponding author: *J. Andrade. E-mail: jelmirandrade@outlook.com ORCID: https://orcid.org/0000-0002-4886-302X and *C. Adam Conte-Junior. E-mail: conte@iq.ufrj.br ORCID: https://orcid.org/0000-0001-6133-5080, Institute of Chemistry, Federal University of Rio de Janeiro, Rio de Janeiro, Brazil.

Analytical Greenness report sheet

17/07/2026 11:35:03

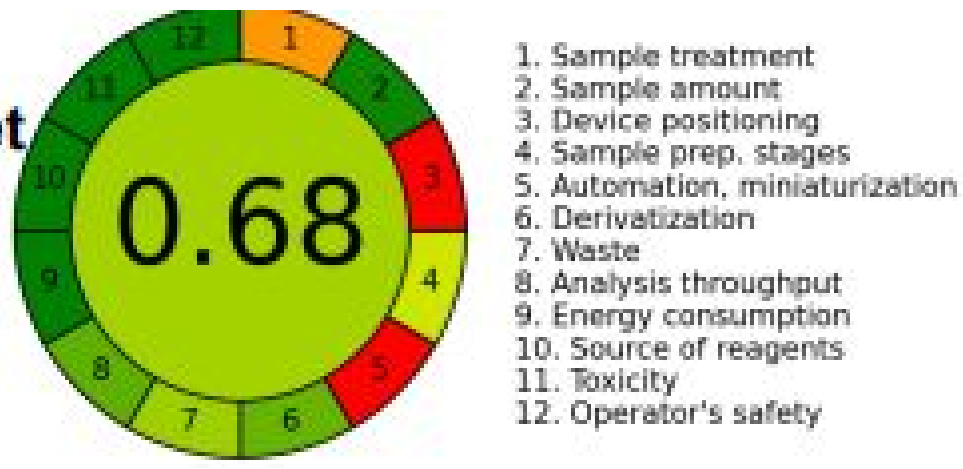

Criteria	Score	Weight
1. Direct analytical techniques should be applied to avoid sample treatment.	0.3	2
2. Minimal sample size and minimal number of samples are goals.	0.98	2
3. If possible, measurements should be performed in situ.	0.0	2
4. Integration of analytical processes and operations saves energy and reduces the use of reagents.	0.6	2
5. Automated and miniaturized methods should be selected.	0.0	2
6. Derivatization should be avoided.	0.8	2
7. Generation of a large volume of analytical waste should be avoided, and proper management of analytical waste should be provided.	0.69	2
8. Multi-analyte or multi-parameter methods are preferred versus methods using one analyte at a time.	0.82	2
9. The use of energy should be minimized.	1.0	2
10. Reagents obtained from renewable sources should be preferred.	1.0	2
11. Toxic reagents should be eliminated or replaced.	1.0	2
12. Operator's safety should be increased.	1.0	2

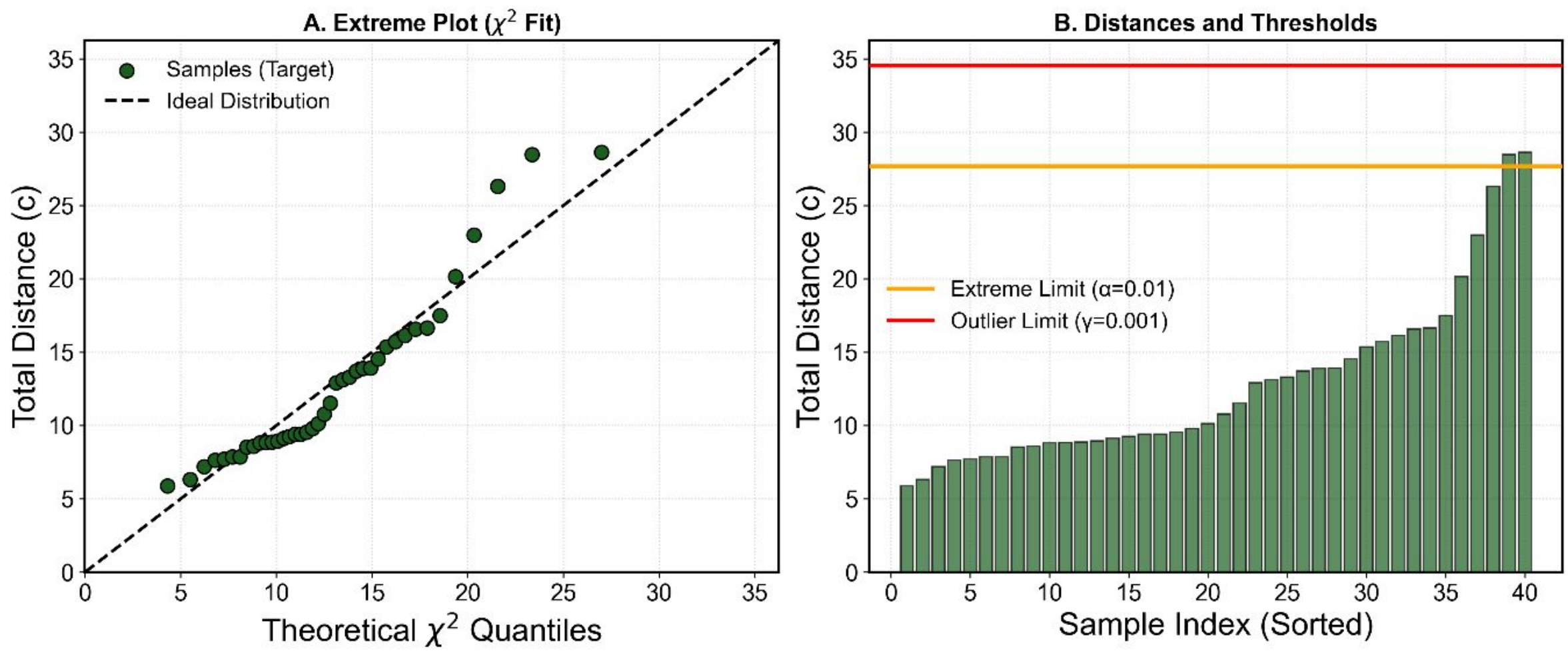

Figure S1. Diagnostic plots of the DD-SIMCA model for the calibration set (authentic guarana samples). (A) Extreme plot of the empirical total distances (c) versus the theoretical quantiles of the chi-square distribution. The dashed black line represents the ideal distribution fit. (B) Sorted total distances for the calibration samples plotted against the theoretical decision thresholds: the extreme limit (orange line, α = 0.01) and the outlier limit (red line, γ = 0.001).

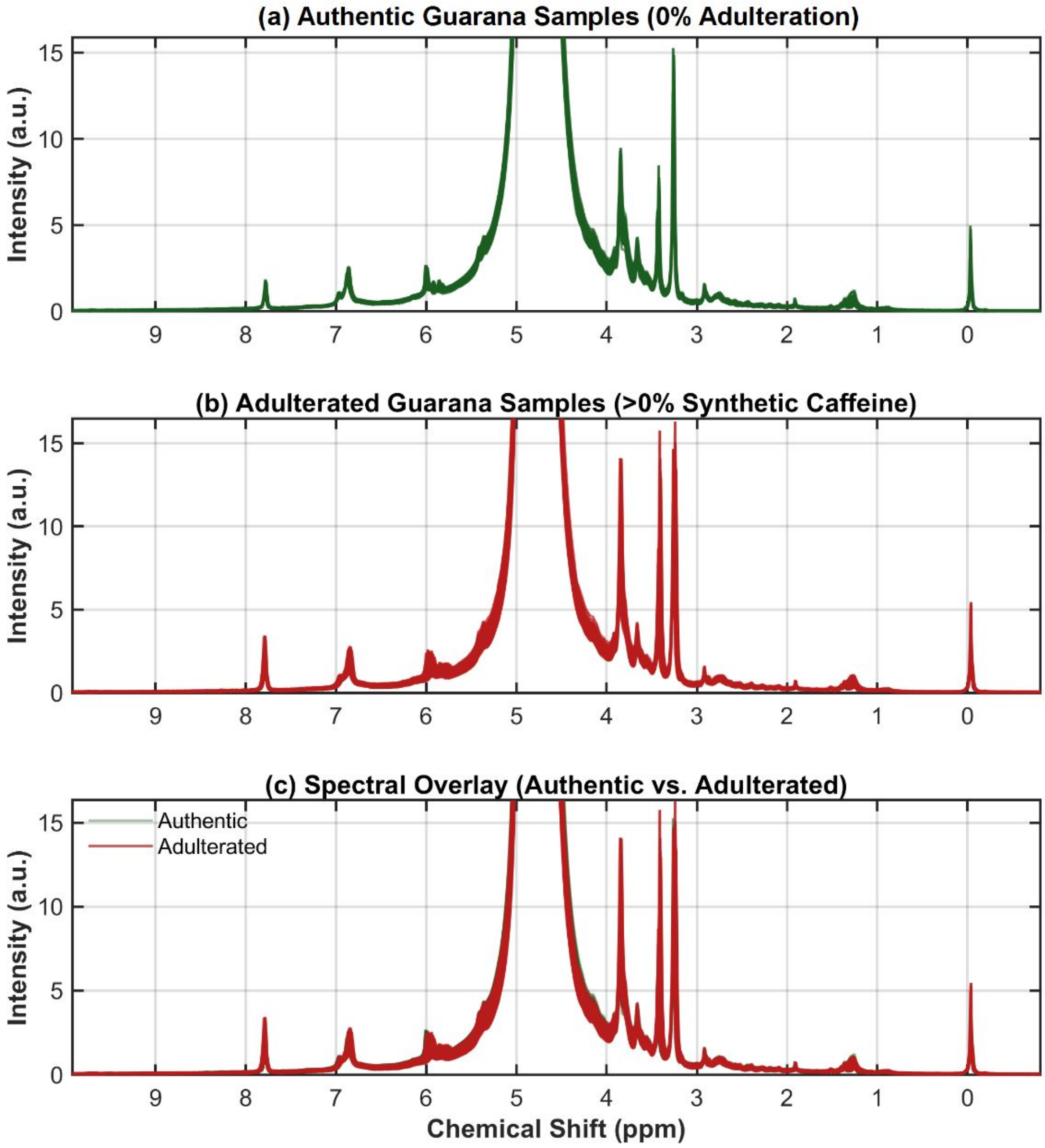

Figure S2. Overlay of the individual LF ^{1}H NMR spectra obtained from powdered guaraná samples. (a) Authentic reference samples (n = 40; green; 0% synthetic caffeine addition). (b) Laboratory-adulterated samples (n = 120; red) containing 1–10% (w/w) added synthetic caffeine. (c) Combined spectral overlay of the authentic and laboratory-adulterated sample groups.

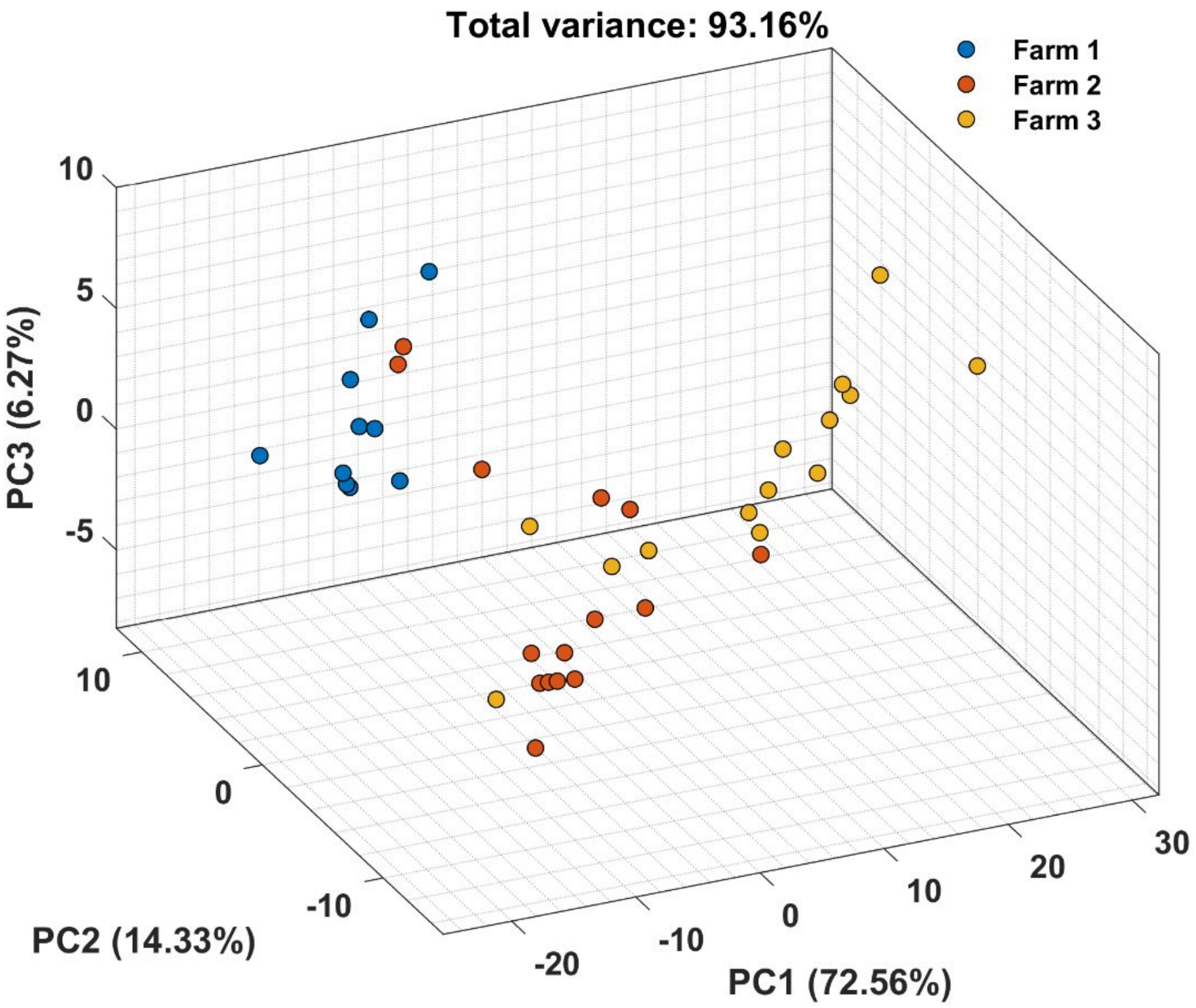

Figure S3. Principal component analysis score plot obtained exclusively from the LF ^{1}H NMR spectra of the 40 authentic, non-adulterated guaraná samples.

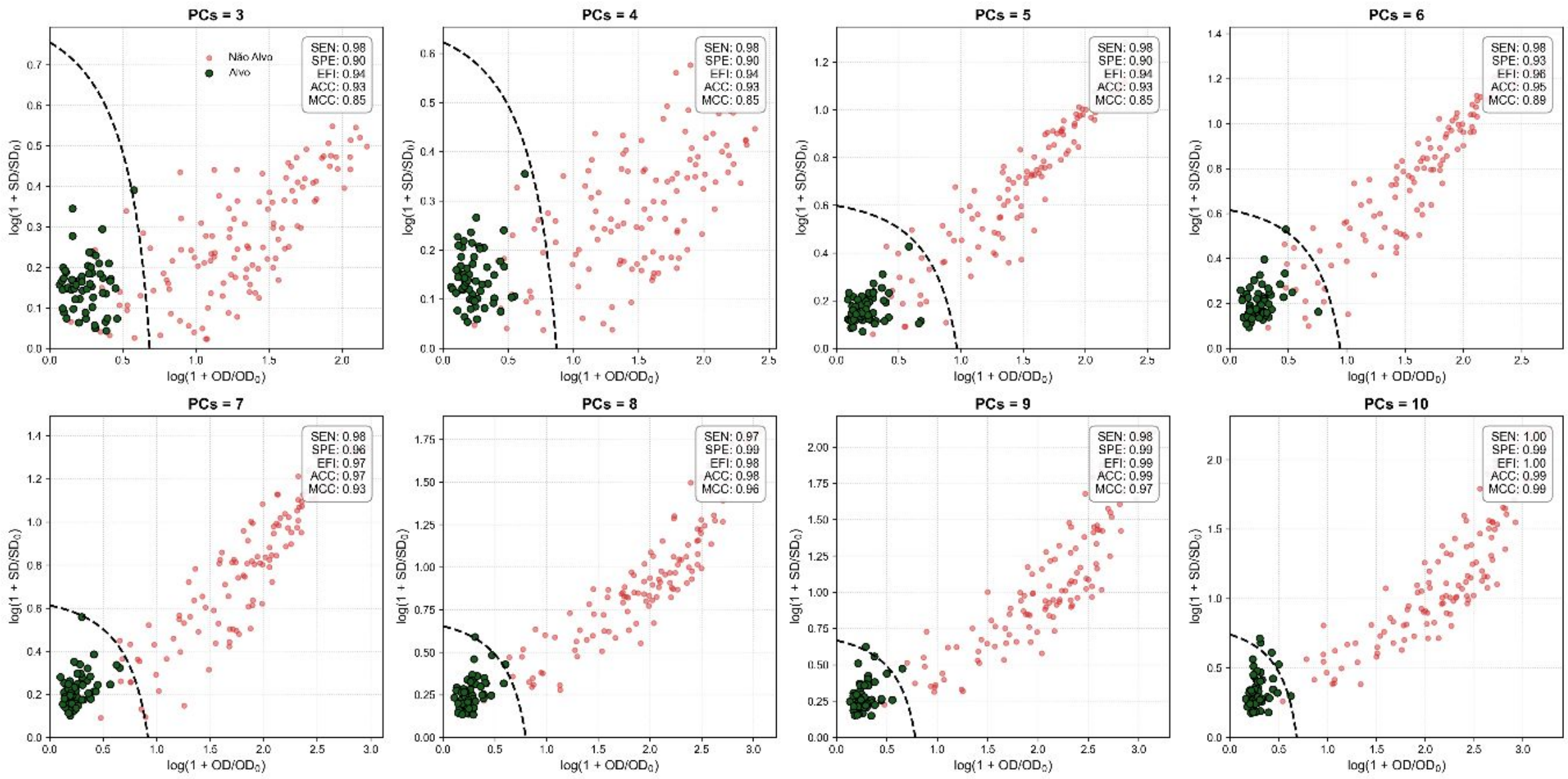

Figure S4. Performance of the DD-SIMCA models constructed with different numbers of Principal Components (PCs = 3–10). For each model, sensitivity (SEN), specificity (SPE), accuracy (ACC), and Matthews correlation coefficient (MCC) are presented.

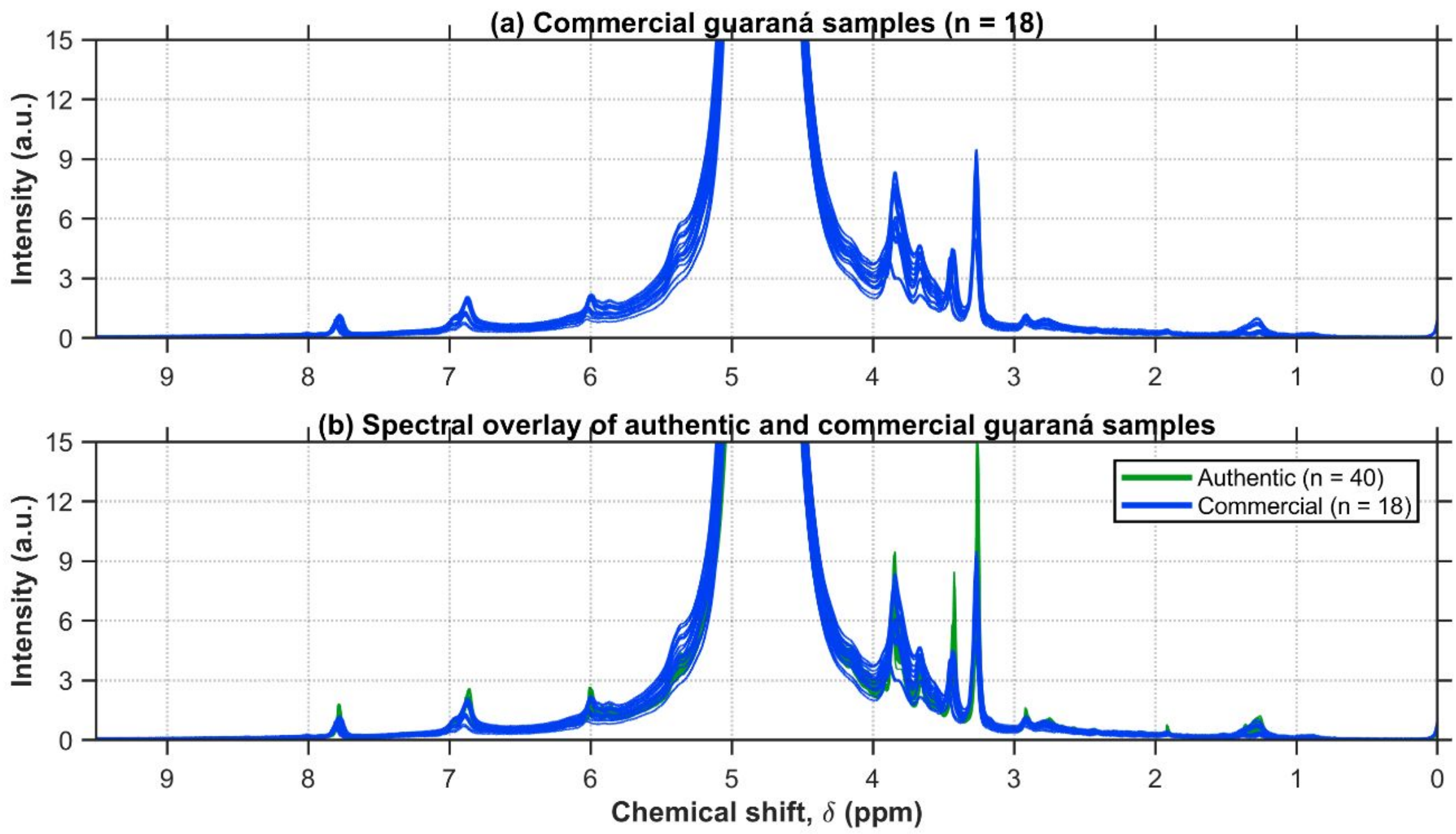

Figure S5. LF ^1H NMR spectral profiles of commercial and authentic powdered guaraná samples. (a) Superimposed individual spectra of the 18 commercial samples. (b) Overlay of the spectra from the 40 authentic reference samples and the 18 commercial samples.

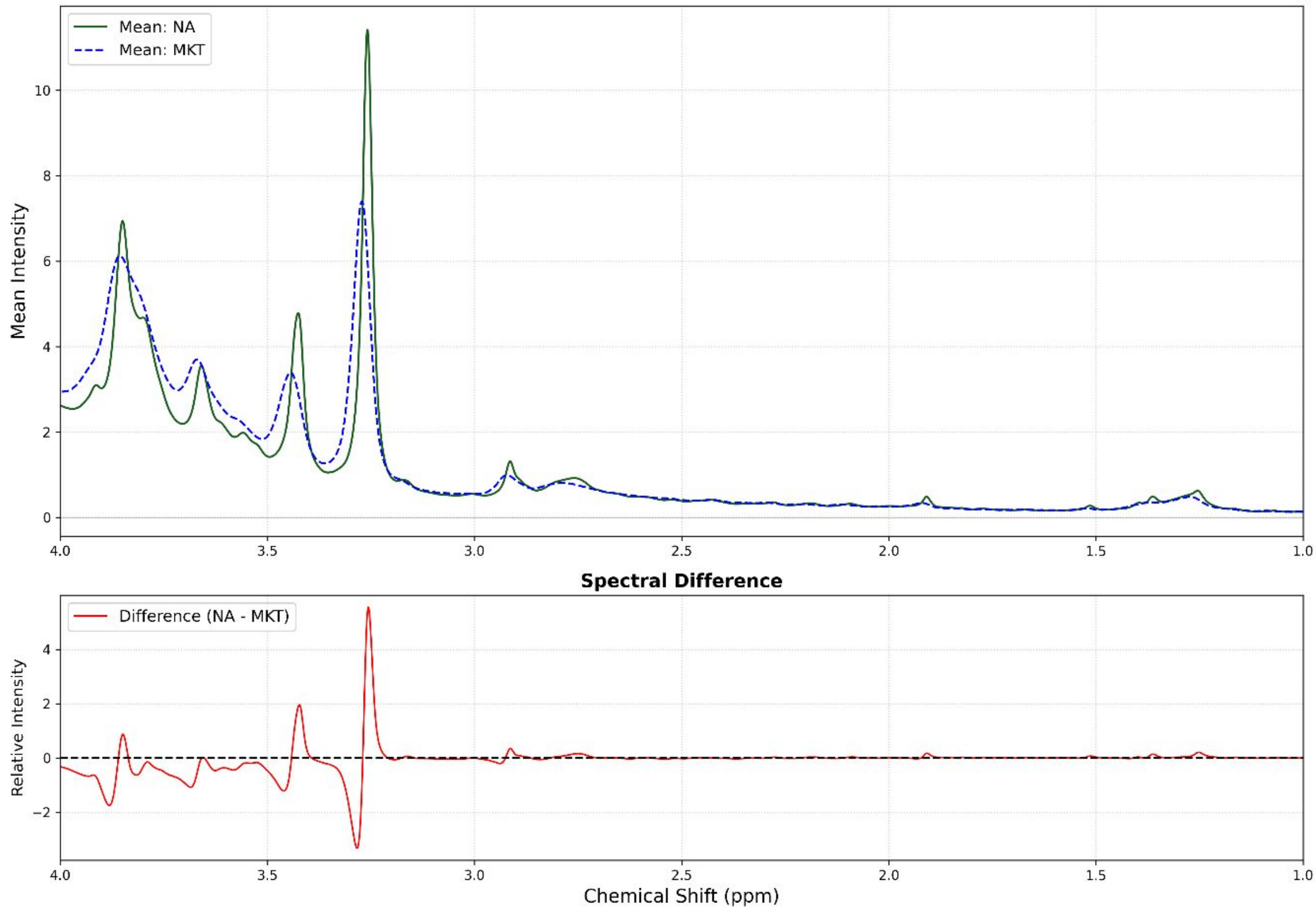

Figure S6. Comparison of the mean LF ^1H NMR spectra between authentic guarana samples (NA) and commercial market samples (MKT). (Top) Overlay of the average spectral profiles for authentic samples (green solid line) and commercial samples (blue dashed line) in the region of δ 1.0 to 4.0 ppm. (Bottom) Spectral difference plot (NA - MKT, red line).

Table S1. Gravimetric preparation of authentic and synthetic caffeine-adulterated guaraná samples

Sample group	Synthetic caffeine addition (% w/w)	Authentic guaraná powder per preparation (mg)	Homogenized caffeine powder per preparation (mg)	Final solid mass (mg)
**Authentic reference samples**	0	100	0	100
**Laboratory-adulterated samples**	1	99	1	100
	2	98	2	100
	3	97	3	100
	4	96	4	100
	5	95	5	100
	6	94	6	100
	7	93	7	100
	8	92	8	100
	9	91	9	100
	10	90	10	100
**Commercial market samples**	Unknown	100	No experimental addition	100

Note: Synthetic caffeine addition was calculated on a final-mixture-mass basis; therefore, the combined mass of guaraná powder and synthetic caffeine was maintained at 100 mg for all preparations. At each addition level, 12 independent laboratory-adulterated samples were prepared using four authentic samples from each of the three farms, resulting in 120 adulterated samples across the ten levels. Each sample was independently weighed, spiked, extracted, and analyzed.
